# Association of circadian rhythms, MTNR1B, BMAL1, BMAL2, and CRY2 gene polymorphisms and their interactions with type 2 diabetes in coal miners

**DOI:** 10.3389/fendo.2026.1567465

**Published:** 2026-03-02

**Authors:** Qinglin Li, Haoyue Cao, Xiaohong Zhang, Juxiang Yuan, Chunze Zhang

**Affiliations:** 1 Department of Public Health, Tianjin Union Medical Center (First Affiliated Hospital of Nankai University), Tianjin, China; 2 Department of Epidemiology and Health Statistics, School of Public Health, North China University of Science and Technology, Tangshan, Hebei, China; 3 Department of Tuberculosis, Tianjin Hexi District Center for Disease Control, Tianjin, China; 4 Department of Colorectal Surgery, Tianjin Union Medical Center (First Affiliated Hospital of Nankai University), Tianjin, China

**Keywords:** circadian rhythm, gene polymorphism, interaction effect, MINER, type 2 diabetes mellitus

## Abstract

**Objective:**

To investigate the interaction between circadian rhythm disorders and related gene polymorphisms (SNPs) in the risk of developing type 2 diabetes (T2DM).

**Methods:**

Cross-sectional study included 4,070 coal miners who underwent occupational health examinations between 2017 and 2018. We constructed comprehensive indicators of circadian rhythm disorder (CICRD) using factor analysis. In case-control analysis, 424 cases and 464 controls were randomly selected from 3,878 male coal miners. Logistic regression models were used to examine the relationship between CICRD, selected SNPs, and T2DM. Gene-gene and gene-environment interactions were evaluated using log-linear models and the generalized multifactor dimensionality reduction (GMDR) method.

**Results:**

The CICRD captures 79.771% of seven circadian rhythm disorder assessment indicators. Higher CICRD and variants at rs10830963 (MTNR1B), rs7958822 (BMAL2), and rs11605924 (CRY2) were associated with an increased risk of T2DM (*P* < 0.05).A CICRD score ≥ 0.2782 with each high-risk SNP (rs10830963, rs1387153, rs7958822, rs11605924) significantly increased T2DM risk (P < 0.05).The five-factor interaction model (rs10830963-rs7950226-rs7958822-rs11605924-CICRD) based on the GMDR method significantly increased T2DM risk in the full dataset (P < 0.05).

**Conclusion:**

The interaction between circadian rhythm disruption and high-risk SNP genotypes further amplifies the risk of T2DM among coal miners. Notably, the five-factor interaction model (rs10830963-rs7950226-rs7958822-rs11605924-CICRD) provides a standardized basis for assessing circadian rhythm disruption, screening high-risk populations, and identifying that high-risk genetic combinations are unsuitable for shift work, offering scientific evidence for the precision prevention of T2DM in coal miners.

## Background

Diabetes is marked by chronic hyperglycemia from impaired insulin secretion or action (1). In 2021, 536.6 million (10.5%) people aged 20–79 had diabetes globally, projected to rise to 783.2 million (12.2%) by 2045 (2). With the advance in modern biopsychosocial medical models (3), recent researches have focused on the complex interactions of factors in T2DM.

Studies show shift work is linked to T2DM onset and progression (4, 5). Shift work, including rotating, evening, and night shifts, is widely used in China’s mining and manufacturing industries to cover 8–24 hours tasks. In 2007, the International Agency for Research on Cancer classified shift work as a Group 2A carcinogen (6). Shift work disrupts sleep-wake patterns, causing circadian rhythm disturbances and health-impacting disorders (7, 8). In addition to shift work, sleep disorders and insufficient sleep disrupt circadian rhythms, especially when sleep and wakefulness occur at inappropriate biological times (9, 10). Moreover, artificial light at night disrupts sleep cycles and circadian functions, altering hormone production and potentially causing diseases (11, 12). Therefore, in addition to shift work, factors like nighttime light exposure, sleep disorders, and insufficient sleep are crucial in studying circadian rhythm disruption.

In recent years, advancements in genomic technologies have facilitated the discovery of new genes and variants associated with T2DM and its traits. The association between circadian rhythm gene polymorphisms and T2DM has become a research focus. Kan MY et al. (13) found that the G allele of rs10830963 and the T allele of rs1387153 in the MTNR1B gene are linked to higher T2DM risk and elevated FG in Han Chinese and European populations. A Japanese study first identified a significant association between the AG+AA genotype of the BMAL2 gene rs7958822 and T2DM risk in obese individuals (14). Although circadian rhythm gene polymorphisms are significantly associated with T2DM, the risk alleles of certain variants remain controversial. A study in the general Chinese population found that the A allele of the CRY2 gene rs11605924 was significantly positively associated with impaired FG and T2DM (15). Another study in the Saudi Arabian population reported that the A allele of rs11605924 is a protective factor against T2DM risk (16). Genetic backgrounds in different populations may influence the association between circadian rhythm gene polymorphisms and T2DM risk, warranting further investigation across multiple populations to elucidate the underlying mechanisms.

The coal industry is a key pillar of China’s economy and energy supply. Coal miners’ quality of life is a important concern in China’s occupational health. Widespread shift work makes circadian rhythm disruption common among coal miners. This study examines circadian rhythm disruption among Xingtai coal miners in the Beijing-Tianjin-Hebei region as part of a health cohort study. Our study examines environmental and genetic impacts on circadian rhythm, offering evidence for T2DM prevention in coal miners.

## Methods

### Study population

Participants were drawn from the baseline data of the coal miner cohort in the Beijing-Tianjin-Hebei Occupational Health Effects Cohort Study, initiated by China’s Ministry of Science and Technology to investigate the impact of occupational hazards on human health. The study included 4,440 workers from the Xingtai coal mining site who underwent health exams between August 2017 and August 2018. All fasting venous blood samples were collected in the morning within a standardized time window (8:00-10:00 a.m.) to minimize circadian variability. Data were collected from questionnaires, physical examinations, laboratory tests, and assessments of occupational hazards. After excluding individuals with incomplete questionnaires (246), less than one year of work experience (22), missing biochemical (12) or physical exam data (27), or invalid questionnaire information (63), 4,070 Han Chinese coal miners were included in the study. Cross-sectional study found CICRD significantly associated with T2DM risk in male workers, but not in females, likely due to the small sample size (**Supplementary Table 1**). Therefore, based on inclusion and exclusion criteria (**Supplementary Materials**), 3,878 male coal miners were randomly sampled as participants for the case-control study. Due to limited funding for genotyping all participants in the cross-sectional survey, we conducted a case-control study using baseline data to explore the association and interaction between CICRD and circadian rhythm-related gene polymorphisms (SNPs) with T2DM. A total of 424 cases and 464 controls were selected as study participants (**Supplementary Figure 1**).

### Evaluation of T2DM and CICRD

According to the China T2DM Prevention and Control Guideline (2020 Edition) (2), T2DM is defined as fasting blood glucose ≥7.0 mmol/L, random blood glucose ≥11.1 mmol/L, or a previous hospital diagnosis of T2DM. Our study constructed CICRD using seven indicators across shift work, light exposure, and sleep: shift duration, cumulative night shifts, night shift frequency and duration, nighttime light exposure, insomnia status, and average sleep duration. Data was collected via interviews and verified with company records. Detailed definitions are presented in the **Supplementary Materials**.

### Definition and classification of covariates

The study included demographic data (gender, age, education, marital status, family income), lifestyle behaviors (smoking, drinking, physical activity, diet), medical history (central obesity, hypertension, liver dysfunction, dyslipidemia, renal dysfunction), and occupational exposures (dust, heat, CO, noise), with detailed definitions in the **Supplementary Materials**.

### Genetic testing of MTNR1B, BMAL1, and BMAL2

Genomic DNA was extracted from whole blood using a Genesky kit, and its concentration and purity were assessed with a spectrophotometer. Tag SNPs related to circadian rhythm genes were selected based on a minor allele frequency (MAF) ≥10% in the Chinese population and a linkage disequilibrium (LD) coefficient >0.8. The Tagger algorithm in HaploView 4.2 was used for selection, prioritizing SNPs identified by genome-wide association studies (GWAS) as linked to T2DM. Six SNPs were selected: rs10830963 and rs1387153 (MTNR1B), rs11022775 and rs7950226 (BMAL1), rs7958822 (BMAL2), and rs11605924 (CRY2). Detailed detection methods are provided in the **Supplementary Tables S2**-**S8**.

### Establishment of CICRD

The suitability of the data for factor analysis was confirmed using the KMO test (0.774) and Bartlett’s sphericity test (*P* < 0.001). CICRD was developed based on seven indicators, including shift duration, nighttime light exposure, and sleep status. Principal component analysis identified three factors: F1 (Shift Work Factor), F2 (Sleep Factor), and F3 (Light Exposure Factor). Factor weights were assigned based on explanatory variance, and the final CICRD score was normalized, explaining 79.711% of the data. This score serves as a quantitative tool to explore the link between circadian rhythm disruption and T2DM. Details are provided in the **Supplementary Tables S9**-**S12**.

### Statistical analysis

Continuous variables were presented as mean ± standard deviation (SD) or median with interquartile range (IQR) and analyzed using t-tests or Mann-Whitney U tests for group comparisons. Categorical variables were presented as frequency (n) and percentage (%) and analyzed using chi-square tests, Fisher’s exact tests, or Cochran-Armitage trend tests for group comparisons. Logistic regression model was performed to explore the association between influencing factors and T2DM. Factor analysis constructed the CICRD, and a restricted cubic spline (RCS) function modeled the dose-response relationship between CICRD and T2DM, with knots at the 5th, 35th, 65th, and 95th percentiles. The Hardy-Weinberg equilibrium (HWE) test was used to determine whether the control group was a random sample from the target population. Logistic regression combined with SNPStats software was used to analyze the association of target SNPs with T2DM under codominant, dominant, recessive, over dominant, and additive models among male coal miners. The optimal model was selected based on the Akaike Information Criterion (AIC) and Bayesian Information Criterion (BIC). The median was used to classify CICRD into “<0.2782” and “≥0.2782”. The CICRD and the dominant model of target SNPs were cross-classified, and logistic regression with multiplicative interaction and the Andersson additive interaction model were used to analyze gene-gene and gene-environment interactions. GMDR 0.9 was used for higher-order interaction analysis to construct optimal gene-gene and gene-environment interaction models. A two-tailed test with a significance level of α = 0.05 was applied.

## Result

### Demographic characteristics of participants in cross-sectional and case-control studies

Cross-sectional study included 4,070 participants (3,878 males, 192 females). Males were older (39.41 ± 8.62 years) than females (36.52 ± 8.81 years), and type 2 diabetes prevalence was higher in males (16.7%) than in females (12.5%) (**Table 1**). Case-control study showed a higher CICRD score in case group (0.33 ± 0.15) than control group (0.29 ± 0.14), with significant differences in age, income, smoking, dyslipidemia, liver dysfunction, hypertension, obesity, diabetes family history, and noise exposure (P < 0.05) (**Supplementary Table 13**).

**Table 1:** Sociodemographic characteristics of the subjects in the cross-sectional study.

Variable	Total population (n=4070)	Male (n=3878)	Female (n=192)	*P*
Age (years)	39.27 ± 8.65	39.41 ± 8.62	36.52 ± 8.81	<0.001^a^
DASH score	23.29 ± 2.77	23.28 ± 2.69	23.56 ± 3.98	<0.001^a^
Per capita monthly household income (Yuan/person)	1887.00 (1509.60,2516.00)	1887.00 (1509.60,2516.00)	1943.50 (1687.50,2516.00)	<0.001^a^
average sleep duration (h/d)	7.37 ± 1.29	7.36 ± 1.30	7.56 ± 1.19	0.020^a^
duration of shift work (year)	9.34 (0.37, 15.59)	9.42 (0.00, 14.93)	7.51 (2.08, 24.44)	0.002^a^
cumulative number of night shifts (night)	494 (21, 1171)	494 (0, 1134)	772 (253, 2229)	<0.001^a^
cumulative duration of night shifts (h)	3621 (131,8643)	3595 (0,8080)	6178 (2029,18033)	<0.001^a^
average frequency of night shifts (nights/month)	3.80 (3.04, 7.60)	3.80 (0.00, 7.60)	7.60 (6.08, 10.14)	<0.001^a^
Marital status, *n* (%)				<0.001^a^
Unmarried	165 (4.1)	139 (3.6)	26 (13.5)	
Married	3905 (95.9)	3739 (96.4)	166 (86.5)	
Education level, *n* (%)				<0.001^c^
Primary level	49 (1.2)	48 (1.2)	1 (0.5)	
Intermediate level	2922 (71.8)	2822 (72.8)	100 (52.1)	
Advanced level	1099 (27.0)	1008 (26.0)	91 (47.4)	
Smoking status, *n* (%)				<0.001^c^
Never smoking	1607 (39.5)	1440 (37.1)	167 (87.0)	
Ever smoking	298 (7.3)	297 (7.7)	1 (0.5)	
Current smoking	2165 (53.2)	2141 (55.2)	24 (12.5)	
Drinking status, *n* (%)				<0.001^c^
Never drinking	953 (23.4)	775 (20.0)	178 (92.7)	
Ever drinking	192 (4.7)	192 (5.0)	0 (0.0)	
Current drinking	2925 (71.9)	2911 (75.0)	14 (7.3)	
Salt taste preference, *n* (%)				0.575^b^
Light	711 (17.5)	677 (17.5)	34 (17.7)	
Moderate	1955 (48.0)	1869 (48.2)	86 (44.8)	
Salty	1404 (34.5)	1332 (34.3)	72 (37.5)	
Insomnia status, *n* (%)				0.064^b^
No sleep disorder	2776 (68.2)	2655 (68.5)	121 (63.0)	
Suspected insomnia	861 (21.2)	820 (21.1)	41 (21.4)	
Insomnia	433 (10.6)	403 (10.4)	30 (15.6)	
Physical activity level, *n* (%)				<0.001^b^
Low	557 (13.7)	414 (10.7)	143 (74.5)	
Moderate	1647 (40.5)	1631 (42.0)	16 (8.3)	
High	1866 (45.8)	1833 (47.3)	33 (17.2)	
Family History of Diabetes, *n* (%)				0.006
No	3565 (87.6)	3409 (87.9)	156 (81.2)	
Yes	505 (12.4)	469 (12.1)	36 (18.8)	
Central Obesity, *n* (%)				<0.001
No	3015 (74.1)	2849 (73.5)	166 (86.5)	
Yes	1055 (25.9)	1029 (26.5)	26 (13.5)	
Dyslipidemia, *n* (%)				<0.001
No	3038 (74.6)	2872 (74.1)	166 (86.5)	
Yes	1032 (25.4)	1006 (25.9)	26 (13.5)	
Abnormal liver function, *n* (%)				<0.001
No	3268 (80.3)	3089 (79.7)	179 (93.2)	
Yes	802 (19.7)	789 (20.3)	13 (6.8)	
Abnormal Kidney Function, *n* (%)				<0.001
No	3869 (95.1)	3725 (96.1)	144 (75.0)	
Yes	201 (4.9)	153 (3.9)	48 (25.0)	
Hypertension, *n* (%)				<0.001
No	2627 (64.5)	2457 (63.4)	170 (88.5)	
Yes	1443 (35.5)	1421 (36.6)	22 (11.5)	
T2DM, n (%)				<0.001
No	3397 (83.5)	3229 (83.3)	168 (87.5)	
Yes	673 (16.5)	649 (16.7)	24 (12.5)	
Heat exposure, *n* (%)				<0.001
No	377 (9.3)	317 (8.2)	60 (31.2)	
Yes	3693 (90.7)	3561 (91.8)	132 (68.8)	
Noise exposure, *n* (%)				<0.001
No	1878 (46.1)	1849 (47.7)	29 (15.1)	
Yes	2192 (53.9)	2029 (52.3)	163 (84.9)	
Dust exposure, *n* (%)				0.008
No	1238 (30.4)	1196 (30.8)	42 (21.9)	
Yes	2832 (69.6)	2682 (69.2)	150 (78.1)	
CO exposure, *n* (%)				<0.001
No	939 (23.1)	861 (22.2)	78 (40.6)	
Yes	3131 (76.9)	3017 (77.8)	114 (59.4)	
Nighttime light exposure, *n* (%)				0.017^b^
Darkest	1259 (30.9)	1203 (31.0)	56 (29.2)	
Moderate	2047 (50.3)	1962 (50.6)	85 (44.3)	
Brightest	764 (18.8)	713 (18.4)	51 (26.5)	
Shift work, n (%)				<0.001
No	1006 (24.7)	986 (25.4)	20 (10.4)	
Yes	3064 (75.3)	2892 (74.6)	172 (89.6)	

^a^ indicates that the Mann-Whitney U test was used due to non-compliance with parametric test assumptions; ^b^ denotes the result obtained by the Cochran-Armitage trend test; ^c^ refers to the Fisher’s exact test. Continuous variables are presented as mean ± SD or median (lower quartile, upper quartile).

### Analysis of association between CICRD and T2DM

**Supplementary Figure 2** shows a positive linear relationship between CICRD and the risk of T2DM among coal miners (*P*_for overall association_<0.001; *P*_for non-linearity_=0.524).Coal miners with CICRD scores of “0.2782–” and “≥0.3848” have 1.43-fold (95% CI: 1.07–1.90) and 2.43-fold (95% CI: 1.80–3.21) higher T2DM risks, respectively, compared to those with scores below “0.1839” (**Table 2**). Sensitivity analysis confirmed the association between CICRD and T2DM across subgroups, consistent with the main findings (**Supplementary Table 1**).

**Table 2:** Logistic regression analysis of CICRD and T2DM in coal miners.

CICRD	n, (%)	OR (95% CI)		
		Model 1	Model 2	Model 3
<0.1839	1017 (24.99)	1.00	1.00	1.00
0.1839~	1017 (24.99)	1.07 (0.82~1.40)	1.15 (0.85~1.54)	1.06 (0.78~1.43)
0.2782~	1019 (25.03)	1.42 (1.10~1.82)	1.51 (1.14~2.02)	1.43 (1.07~1.90)
≥0.3848	1017 (24.99)	2.26 (1.77~2.89)	2.38 (1.79~3.17)	2.43 (1.80~3.21)
Test for trend		1.33 (1.22~1.44)	1.34 (1.22~1.47)	1.35 (1.23~1.48)
Per *SD* increase		1.40 (1.28~1.52)	1.39 (1.26~1.54)	1.41 (1.28~1.57)

Model 1: adjusted for age and gender;.

Model 2: further adjusted for marital status, family income per capita, education level, smoking status, drinking status, salt taste preference, physical activity level, DASH score, abnormal liver function, abnormal renal function, dyslipidemia, hypertension, and family history of diabetes;

Model 3: further adjusted for occupational hazards (CO, noise, dust and heat);

SD: standard deviation.

### Correlation and interaction between targeted SNPs and T2DM in coal miners

**Supplementary Table 14** indicates that the dominant model is optimal for rs10830963, rs7958822, and rs11605924, with CG/GG (*OR* = 1.50, 95% *CI*: 1.14–1.98), GA/AA (*OR* = 1.43, 95% *CI*: 1.09–1.86), and AC/CC (*OR* = 1.35, 95% *CI*: 1.03–1.76) genotypes, respectively, showing a higher T2DM risk compared to their reference genotypes. However, rs1387153 (MTNR1B), rs11022775 (BMAL1), and rs7950226 (BMAL1) showed no statistically significant association with T2DM under any genetic model. Based on above findings, three T2DM susceptibility loci were identified, and their interactions were analyzed using cross-classification under dominant models. **Supplementary Table 15** shows that T2DM risk significantly increases when rs10830963 CG+GG is combined with rs7958822 GA+AA (*OR* = 2.71, 95% *CI*: 1.77–4.15), rs11605924 AC+CC (*OR* = 2.64, 95% *CI*: 1.70–4.11), or when rs7958822 GA+AA is combined with rs11605924 AC+CC (*OR* = 2.77, 95% *CI*: 1.80–4.27), compared to their respective reference genotypes. In addition, there were positive interactions between rs10830963 and rs11605924, rs7958822 and rs11605924 on T2DM (*P* < 0.05).

GMDR analysis showed that the four-factor model (rs10830963-rs1387153-rs7958822-rs11605924) was statistically significant (P = 0.003) and achieved a cross-validation consistency of 91.67% (11/12), making it the optimal high-order interaction model (**Supplementary Table 16**). In this model, the high-risk group is marked in dark gray (**Supplementary Figure 3**). In the full dataset, coal miners with homozygous mutations had a 3.10-fold (95% *CI*: 2.01–4.77) higher risk of T2DM compared to those with wild-type genotypes (**Supplementary Table 17**).

**Figure 1:**
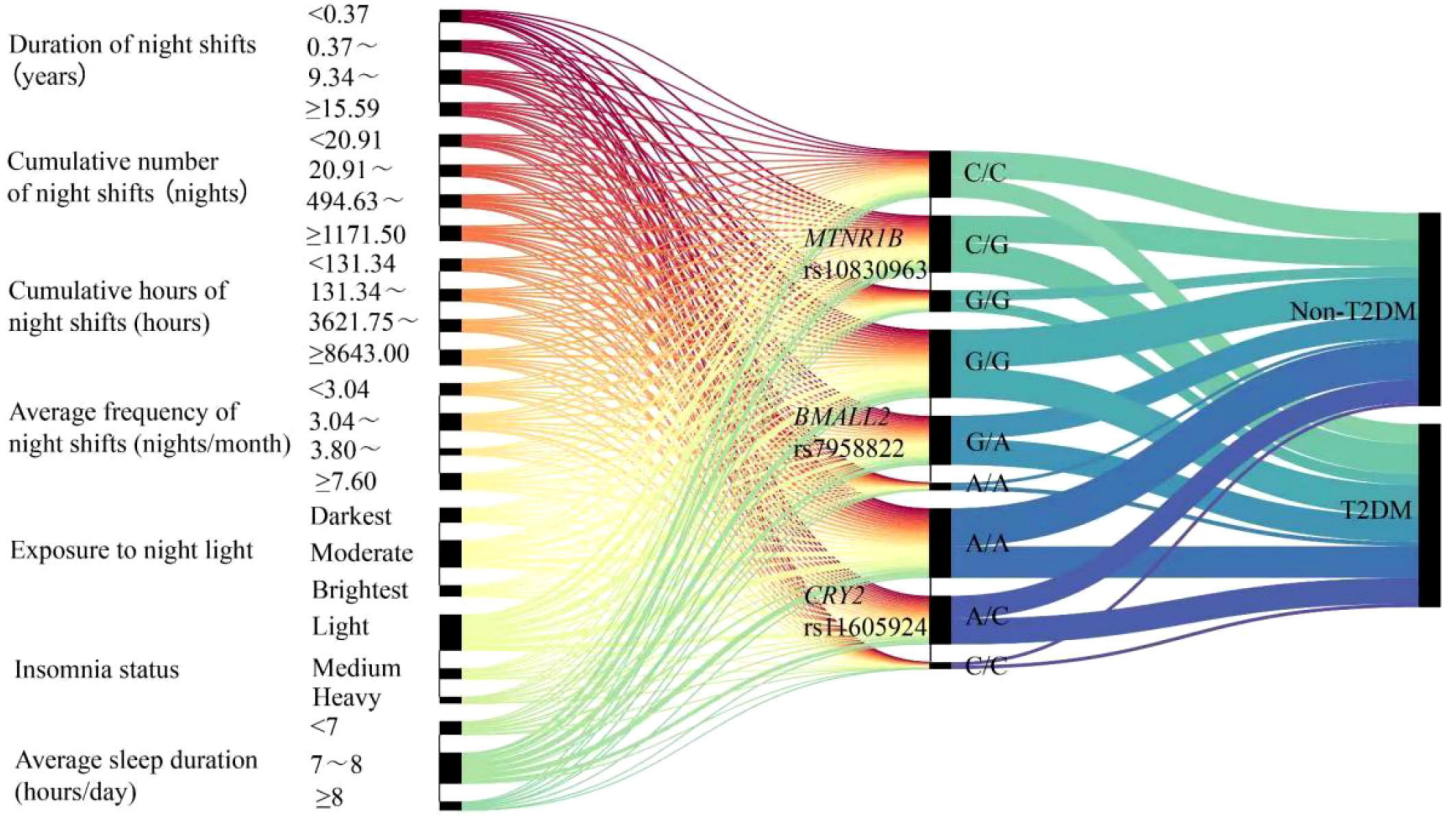
Sankey diagram of the interaction between environmental and genetic factors.

On the left side of the Sankey diagram were the different levels of indicators related to circadian rhythm disorder, among which were the genotypes of three biological clock genes, and on the right side were the dichotomous outcomes (T2DM/non-T2DM); The line width corresponds to the number of people in the combination of “environmental factors+genotype”, which intuitively showed the impact of environmental genetic interaction on T2DM. The combination of long-term, high-frequency night shifts, severe insomnia, and short sleep environments with high exposure levels, combined with the G/G genotype of MTNR1B, the A/A genotype of BMAL2, and the C/C genotype of CRY2, corresponds to a thicker line flowing towards “T2DM”, which is a high-risk combination of T2DM (**Figure 1**).

### Analysis of interaction between CICRD and various gene loci

CICRD was categorized as “<0.2782” or “≥0.2782” and analyzed for SNP interactions. The risk of T2DM is significantly higher for CICRD ≥ 0.2782 combined with specific genotypes: rs10830963 CG+GG (*OR* = 2.67, 95% *CI*: 1.77–4.15), rs1387153 CT+TT (*OR* = 1.93, 95% *CI*: 1.30–2.87), rs7958822 GA+AA (*OR* = 2.29, 95% *CI*: 1.60–3.27), and rs11605924 AC+CC (*OR* = 2.30, 95% *CI*: 1.63–3.25), compared to CICRD < 0.2782 combined with their respective reference genotypes. However, our study found no significant additive or multiplicative interactions between CICRD and individual gene loci (**Table 3**).

**Table 3:** Multiplicative and additive interactions of shift work and genes on T2DM risk.

SNPs	CICRD	Genotype	Cases	Controls	*OR* (95% *CI*)		*P*_Multiplicative Interaction_	
					Model 1	Model 2	Model 1	Model 2
rs10830963	<0.2782	CC	51	103	1.00	1.00	0.719	0.614
		CG+GG	121	135	1.81 (1.19~2.74)	2.16 (1.37~3.46)		
	≥0.2782	CC	89	88	2.04 (1.31~3.19)	2.00 (1.22~3.30)		
		CG+GG	171	130	2.66 (1.77~3.99)	2.67 (1.69~4.22)		
	Trend test				1.32 (1.17~1.50)	1.29 (1.13~1.48)		
	RERI				-0.20 (-1.25~0.86)	-0.50 (-1.76~0.75)		
	AP				-0.07 (-0.47~0.33)	-0.19 (-0.66~0.28)		
rs1387153	<0.2782	CC	63	95	1.00	1.00	0.968	0.743
		CT+TT	109	143	1.15 (0.77~1.72)	1.48 (0.94~2.33)		
	≥0.2782	CC	96	86	1.68 (1.09~2.59)	1.76 (1.08~2.84)		
		CT+TT	164	132	1.87 (1.27~2.77)	1.93 (1.24~3.00)		
	Trend test				1.25 (1.11~1.41)	1.22 (1.06~1.40)		
	RERI				0.04 (-0.74~0.82)	-0.30 (-1.27~0.66)		
	AP				-0.02 (-0.39~0.44)	-0.16 (-0.65~0.34)		
rs11022775	<0.2782	CC	137	195	1.00	1.00	0.930	0.852
		CT+TT	35	43	1.16 (0.71~1.90)	0.98 (0.57~1.68)		
	≥0.2782	CC	205	178	1.64 (1.22~2.21)	1.44 (1.03~2.03)		
		CT+TT	55	40	1.96 (1.23~3.11)	1.52 (0.91~2.53)		
	Trend test				1.27 (1.12~1.43)	1.18 (1.03~1.36)		
	RERI				0.16 (-0.87~1.19)	0.09 (-0.83~1.01)		
	AP				0.08 (-0.42~0.59)	0.06 (-0.53~0.65)		
rs7950226	<0.2782	AA	66	75	1.00	1.00	0.326	0.095
		GA+GG	106	163	0.74 (0.49~1.12)	0.66 (0.42~1.04)		
	≥0.2782	AA	92	76	1.38 (0.88~2.16)	1.04 (0.63~1.72)		
		GA+GG	168	142	1.34 (0.90~2.00)	1.17 (0.75~1.82)		
	Trend test				1.19 (1.06~1.35)	1.14 (0.99~1.34)		
	RERI				0.23 (-0.38~0.84)	0.46 (-0.06~0.98)		
	AP				0.17 (-0.29~0.63)	0.40 (-0.09~0.88)		
rs7958822	<0.2782	GG	66	75	1.00	1.00	0.937	0.838
		GA+AA	106	163	1.44 (0.97~2.14)	1.77 (1.14~2.75)		
	≥0.2782	GG	92	76	1.67 (1.16~2.40)	1.54 (1.02~2.33)		
		GA+AA	168	142	2.25 (1.55~3.25)	2.29 (1.52~3.48)		
	Trend test				1.30 (1.15~1.46)	1.28 (1.12~1.46)		
	RERI				0.14 (-0.73~1.01)	-0.02 (-1.04~1.00)		
	AP				0.06 (-0.32~0.44)	-0.01 (-0.45~0.44)		
rs11605924	<0.2782	AA	86	136	1.00	1.00	0.838	0.781
		AC+CC	86	102	1.33 (0.90~1.98)	1.47 (0.96~2.27)		
	≥0.2782	AA	138	134	1.63 (1.14~2.33)	1.44 (0.96~2.16)		
		AC+CC	122	84	2.30 (1.56~3.38)	2.30 (1.49~3.56)		
	Trend test				1.31 (1.16~1.48)	1.28 (1.12~1.47)		
	RERI				0.34 (-0.52~1.19)	0.39 (-0.54~1.33)		
	AP				0.15 (-0.21~0.50)	0.17 (-0.21~0.55)		

Adjusted for age, marital status, per capita monthly household income, education level, smoking status, drinking status, salt taste preference, physical activity level, DASH score, liver function abnormalities, kidney function abnormalities, dyslipidemia, hypertension, family history of diabetes, CICRD, and occupational hazards (CO, noise, dust, and heat).

GMDR analysis showed that the five-factor model (rs10830963-rs7950226-rs7958822-rs11605924-CICRD) was statistically significant (P = 0.003) with a cross-validation consistency of 100% (12/12), making it the optimal high-order interaction model (**Table 4**). Five-factor model was selected as the best gene-gene higher-order interaction model, with high-risk combinations shown in **Supplementary Figure 4**. In the full dataset, coal miners with homozygous mutant genotypes combined with CICRD ≥ 0.2782 is 7.38 times (95% CI: 4.84–11.25) higher risk of T2DM compared to those with homozygous wild-type genotypes combined with CICRD < 0.2782 (**Supplementary Table 18**).

**Table 4:** CICRD-Target SNPs interaction models identified by GMDR.

Model	Training set accuracy	Validation set accuracy	*P*	Cross-validation consistency
CICRD	0.5620	0.5438	10 (0.0193)	11/12
rs7950226- CICRD	0.5703	0.5421	8 (0.1938)	10/12
rs7958822- rs11605924- CICRD	0.5953	0.5271	7 (0.3872)	6/12
rs10830963- rs7958822- rs11605924- CICRD	0.6260	0.5357	10 (0.0193)	5/12
rs10830963- rs7950226- rs7958822- rs11605924- CICRD	0.6768	0.5759	11 (0.0032)	12/12
rs10830963- rs1387153- rs7950226- rs7958822- rs11605924- CICRD	0.7073	0.5363	8 (0.1938)	7/12
rs10830963- rs1387153- rs11022775- rs7950226- rs7958822- rs11605924- CICRD	0.7353	0.5354	7 (0.3872)	12/12

Adjusted for age, marital status, per capita monthly household income, education level, smoking status, drinking status, salt taste preference, physical activity level, DASH score, liver function abnormalities, kidney function abnormalities, dyslipidemia, hypertension, family history of diabetes, CICRD, and occupational hazards (CO, noise, dust, and heat). Cross-Validation: Refers to randomly dividing the data into 12 parts, using one part as the validation set and the remaining 11 parts as the training set, followed by training and validating the model to ensure balanced testing.

## Discussion

In our study, the CICRD constructed using factor analysis based on seven indicators captured 79.771% of the information from the original data. The CICRD and the gene variants rs10830963 (MTNR1B), rs7958822 (BMAL2), and rs11605924 (CRY2) are significantly associated with the risk of T2DM in coal miners. Notably, both the gene-gene four-factor interaction model (rs10830963-rs1387153-rs7958822-rs11605924) and the CICRD-gene five-factor interaction model (rs10830963-rs7950226-rs7958822-rs11605924-CICRD) are significantly associated with the risk of T2DM in coal miners.

In our study, the assessment indicators related to circadian rhythm disorder include number of years working night shifts, cumulative number of night shifts, total duration of night shifts, average frequency of night shifts, nighttime light exposure, insomnia status, and average sleep duration. However, constructing new indicators involves key challenges with weight allocation. To preserve the original information and minimize subjectivity, this process must be approached carefully. This study used factor analysis to extract common factors from numerous original variables and condense them. Based on the field database, we selected seven fundamental indicators related to shift work, night-time light exposure, and sleep to construct the CICRD. We aimed to explore the association between circadian rhythm disorder and T2DM in coal miners from a comprehensive perspective. The results of this study indicate a positive linear association between CICRD and T2DM in coal miners. The results suggest that reducing the intensity and frequency of shift work, improving sleep quality, and avoiding sleep deprivation and night-time light exposure can help reduce circadian rhythm disorder, which is beneficial for preventing T2DM in coal miners. CICRD provides a basis and standard for assessing circadian rhythm disruption in the coal industry and potentially other industries, while also offering scientific evidence for screening high-risk T2DM populations. Thus, the development, application, and extrapolation of CICRD have significant scientific and public health value.

MTNR1B belongs to the G protein-coupled receptor family involved in insulin secretion and encodes the melatonin receptor 1B (17). Previous studies identified MTNR1B loci associated with fasting glucose (FG). A study based on a European population found that rs1387153 is associated with elevated FG, with the T allele being a risk factor for elevated FG levels and T2DM (18). However, a study based on a Chinese Han population found that the T allele was associated only with FG and had no significant association with T2DM (13). Our results also show that the T allele is not significantly associated with T2DM. The rs10830963 locus is located in the only intron of MTNR1B. A meta-analysis found the rs10830963 G allele linked to higher fasting glucose and reduced β-cell function (19). Additionally, the rs10830963 G allele is a risk factor for elevated FG and T2DM in different populations such as American white people (20), and Han Chinese (21). These findings are consistent with our study results. Melatonin, a hormone from the pineal gland, regulates circadian rhythms (22). Animal studies have demonstrated that melatonin reduces insulin levels (23) and impairs glucose tolerance (24) in rats. These biological mechanisms may help explain the observed associations.

Studies show pancreatic islets have circadian CLOCK and BMAL1 oscillations, and disrupting these components in mice causes hypoinsulinemia and diabetes (25). However, studies on BMAL1 variants rs11022775 and rs7950226 and T2DM are scarce and inconsistent. A study found that haplotypes with the rs11022775 T allele and rs7950226 A allele increase T2DM risk, largely driven by rs11022775 (26). A meta-analysis of 13,781 participants found the BMAL1 rs7950226 A allele associated with reduced metabolic syndrome risk (27). Our study found no significant association between BMAL1 rs11022775 and rs7950226 and T2DM in coal miners, consistent with a study in obese Japanese individuals (14). This indicates the need for larger studies in diverse populations to further explore these relationships.

Animal study suggests that BMAL2 gene expression may influence glucose and insulin levels (28). Previous research on the association between BMAL2 and T2DM is limited. BMAL2 SNPs are linked to psychiatric disorders but show no association with metabolic syndrome in European populations (29, 30). However, a study in an Asian population found the BMAL2 rs7958822 A allele significantly associated with T2DM in obese individuals (14). This aligns with the T2DM risk alleles identified in our study. Genetic susceptibility may vary by ethnicity, warranting further research on BMAL2’s link to T2DM across diverse populations.

The core clock is regulated by transcription factors, with CRY2 playing a key role as a transcriptional repressor. A study in a Chinese population found the CRY2 rs11605924 A allele linked to impaired FG and T2DM (15), contrasting with our findings. However, some evidence supports our findings. A study in a Saudi Arabian population found the rs11605924 A allele protective against T2DM (16). A Chinese study found the rs11605924 C allele to be a T2DM risk allele, also supporting our findings (31).

Beyond their established roles in circadian regulation and glucose metabolism, these genes are embedded in neural networks that govern feeding behavior. Recent reviews have shown that circadian clocks regulate both homeostatic and hedonic food intake via hypothalamic and mesolimbic reward circuits, providing a mechanistic link between circadian misalignment, overeating, and metabolic disease (32, 33). Furthermore, variants in circadian rhythm genes may also influence T2DM risk through their effects on direct physiological markers of circadian rhythmicity. Those genes participate in the regulation of both central and peripheral clocks, and their functional alterations may disrupt 24-hour patterns of blood pressure, core body temperature, and nocturnal melatonin secretion—key physiological markers of internal circadian organization (34–36). Indeed, circadian misalignment has been shown to impair glucose metabolism and insulin sensitivity (37). Studies of circadian rhythm physiology and pancreatic endocrine function further support that clock gene dysregulation can impair insulin secretion and glucose homeostasis (38). Thus, gene-related circadian misalignment may represent an additional mechanistic pathway linking these polymorphisms to metabolic dysfunction and T2DM.

Our study is the first to report a four-factor model (rs10830963-rs1387153-rs7958822-rs11605924) with significant interaction, linking specific genotype combinations to increased T2DM risk. Focusing on genotype combinations, not just individual genes, is crucial for identifying high-risk populations. Previous studies have reported an association between high-dimensional interactions among rs6850524, rs10830963, and rs1387153 loci in the CLOCK gene and metabolic syndrome (39). Lin E et al. (40) found that the interaction between the ARNTL and RORB genes is associated with elevated FG levels.

Circadian rhythm is generated by the combined action of a transcription-translation feedback loop involving interacting clock proteins and external environmental factors. Our study identified a five-factor interaction model (rs10830963-rs7950226-rs7958822-rs11605924-CICRD), where homozygous mutant genotypes combined with CICRD ≥ 0.2782 indicate high T2DM risk in coal miners. A study suggests that in steelworkers, a four-factor combination model (MTNR1A-MTNR1B-CLOCK-shift work) increases the risk of T2DM through complex interactions (41). Another study based on the U.S. Biobank found that the interaction between morning preference and rs10830963 is associated with the risk of T2DM (42). However, the interaction between genes and the environment is complex, and current evidence is insufficient to fully elucidate its mechanisms.

Our study has some limitations. First, the study’s limited female participants showed no significant CICRD-T2DM associations, with findings driven by males, underscoring the need for future large-scale studies on females. Second, self-reported data may cause misclassification bias. Third, the healthy worker effect in this relatively healthy population of Chinese coal miners may limit the generalizability of the conclusions. Fourth, as a cross-sectional study, it cannot establish causal relationships between exposures and outcomes. Finally, the prolonged T2DM development may misclassify pre-T2DM individuals as non-cases, causing selection bias.

## Conclusion

The CICRD captures 79.771% of seven circadian rhythm disorder assessment indicators. Higher CICRD and variants at rs10830963 (MTNR1B), rs7958822 (BMAL2), and rs11605924 (CRY2) were associated with an increased risk of T2DM. The four-factor gene-gene model (rs10830963-rs1387153-rs7958822-rs11605924) and five-factor CICRD-gene model (rs10830963-rs7950226-rs7958822-rs11605924-CICRD) are significantly linked to increased T2DM risk in coal miners. CICRD provides a standard and theoretical basis for evaluating circadian rhythm disruption and identifying high-risk T2DM individuals among coal miners. The identification of T2DM susceptibility genes and their interactions suggests that high-risk combinations are unsuitable for shift work, offering scientific evidence for the precise prevention of T2DM in coal miners.

## Data Availability

The data analyzed in this study is subject to the following licenses/restrictions: Development of the dataset continues. The datasets used and/or analyzed during the current study are available from the corresponding author on reasonable request. Requests to access these datasets should be directed to HC 1113551219@qq.com.
