# Supplementary material for: Association of circadian rhythms, MTNR1B, BMAL1, BMAL2, and CRY2 gene polymorphisms and their interactions with type 2 diabetes in coal miners
Source: Front Endocrinol (Lausanne). 2026 Mar 2;17:1567465. doi: 10.3389/fendo.2026.1567465 (PMC12989341; doi:10.3389/fendo.2026.1567465)
Supplement: Supplementary file 1 [file DataSheet1.docx]

**Supplementary file**

**Association of circadian rhythms, MTNR1B, BMAL1, BMAL2, and CRY2 gene polymorphisms and their interactions with type 2 diabetes in coal miners**

Qinglin Li 1†, Haoyue Cao 2†, Juxiang Yuan 2*, Wenhong Wang 1*

1. Department of Public Health, Tianjin Union Medical Center (First Affiliated Hospital of Nankai University), Tianjin, China;

2. Department of Epidemiology and Health Statistics. School of Public Health, North China University of Science and Technology, No.21 Bohai Avenue, 063210, Tangshan, Hebei Province, China

†Qinglin Li and Haoyue Cao contributed equally to this work

*Corresponding Author:

Juxiang Yuan; No. 21 Bohai Avenue, Caofeidian New City, Tangshan City, Hebei

Province, 063210, ChinaNo. 21 Bohai Avenue, Caofeidian New City, Tangshan City, Hebei Province, 063210, China

Wenhong Wang; Department of Public Health, Tianjin Union Medical Center (First

Affiliated Hospital of Nankai University), Tianjin, China

**inclusion and exclusion criteria**

Inclusion criteria for the case group: ① Han Chinese male coal miners diagnosed with T2DM during the survey; ② a minimum of 3 years of work experience; ③ signed informed consent.

Exclusion criteria for the case group: ① Individuals with severe missing information on shift work, covariates, or blood biochemical data; ② Those taking antiretroviral drugs or diagnosed with cancer or thyroid disease; ③ Individuals with other diseases related to the genes selected in this study.

Inclusion criteria for the control group were: ① Han Chinese male coal miners without T2DM, diagnosed using the same criteria as the case group; ② Age-matched to the case group within ±5 years; ③ A minimum of 3 years of work experience; ④ Comparable residential conditions; ⑤ Signed informed consent.

Exclusion criteria for the control group: ① Individuals with severe missing information on shift work, covariates, or blood biochemical data; ② Those taking antiretroviral medications or diagnosed with cancer or thyroid disorders; ③ Individuals with other diseases related to the genes selected in this study.

Sample size calculation for the case-control study:

$\begin{aligned} n=\frac{\left[ z_{\propto}\sqrt{2\bar{p}\left( 1-\bar{p} \right)}+z_{\beta}\sqrt{p_{1}\left( 1-p_{1} \right)+p_{0}\left( 1-p_{0} \right)} \right]^{2}}{\left( p_{1}-p_{0} \right)^{2}} \end{aligned}$ （6）

$\begin{aligned} n=\frac{\left[ z_{\propto}\sqrt{2\bar{p}\left( 1-\bar{p} \right)}+z_{\beta}\sqrt{p_{1}\left( 1-p_{1} \right)+p_{0}\left( 1-p_{0} \right)} \right]^{2}}{\left( p_{1}-p_{0} \right)^{2}} \end{aligned}$ （7）

$\begin{aligned} \bar{p}=\frac{\left( p_{1}+p_{0} \right)}{2} \end{aligned}$ （8）

$\begin{aligned} p_{1}=\frac{\left( OR\times p_{0} \right)}{\left( 1-p_{0}+OR\times p_{0} \right)} \end{aligned}$ （9）

In the formula:

*P_0_*—Exposure proportion of the research factor in the control group

*P_1_*—Exposure proportion of the research factor in the case group.

The hypothesis test was conducted with a Type I error rate *α*=0.05. In this study, *P_0_* represents the minor allele frequency (MAF) set at 0.101*_._* The expected odds ratio (OR) ranges from 1 to 2. The test power is denoted as Power=1-*β.* A total of 424 cases and 464 controls were included in the study. A sample size and power relationship plot was generated using the PS-Power and Sample Size Calculation software. As shown in Figure S1, the power increases with the sample size. When the number of participants in both the case and control groups reaches at least 424, the power reaches 0.932, exceeding 0.90.

**Assessment of CICRD**

1. Shift work:

(1) Shift Work: Shift work refers to a work schedule which the working hours are not fixed but follow a certain pattern. The primary shift work patterns among coal miners in the Xingmei Group are the four-shift three-operation rotation and the three-shift two-operation rotation. Four-Shift Three-Operation Rotation: In this system, each shift works continuously for 2 days, with 2 morning shifts (8:00 AM–4:00 PM), 2 afternoon shifts (4:00 PM–12:00 AM), 2 night shifts (12:00 AM–8:00 AM), followed by 2 days off within an 8-day cycle. Three-Shift Two-Operation Rotation: This system involves three groups rotating through morning (8:00 AM–4:00 PM), afternoon (4:00 PM–12:00 AM), and night shifts (12:00 AM–8:00 AM), with the rotation occurring weekly. According to the International Labour Organization, night work is defined as work performed between 12:00 AM and 5:00 AM[1].

(2) Shift Work Assessment Indicators:

Due to the complexity of work schedules and environments, the assessment of shift work is a recognized challenge. Simple evaluation metrics often overlook critical information that may impact health, obscuring the true relationship between exposure and outcomes. Therefore, this study utilizes multiple indicators to assess shift work: Duration of Shift Work (years), defined as the total number of years across all types of shift work participated in, with workers having less than one year of shift work classified as non-shift workers; Cumulative number of night shifts (nights), which is the sum of all night shifts worked across different shift patterns; Cumulative duration of night shifts (hours), representing the total hours worked during night shifts in all shift patterns; and average frequency of night shifts (nights/month), calculated as the total number of night shifts divided by the total number of working months.

2. Nighttime light exposure: Assessed based on self-reported information from the study participants, with indoor light intensity during sleep categorized into four levels. Due to the fact that the category "able to read books" represents only 3.7% of the study participants, it was combined with the category "able to see the indoor environment clearly" into a single category defined as "Brightest." The category "able to vaguely discern the indoor environment" was defined as "Moderate," and "almost dark" was defined as "Darkest."

3. Insomnia Status: Evaluated using the Athens Insomnia Scale (AIS) combined with self-reported information from the study participants. In this study, an AIS score of <4 is defined as "No Sleep Disorder"; an AIS score of 4–6 is defined as "Suspected Insomnia"; and an AIS score of >6 is defined as "Insomnia"[2].

4. Average Sleep Duration: Participants reported their daily sleep duration during shift work (morning, afternoon, and night shifts) as well as on non-workdays. The average sleep duration is calculated as a weighted average of sleep times on workdays and non-workdays. For example, during a four-shift, three-rotation schedule, the average sleep duration is calculated as [(Morning shift sleep duration×2)+(Afternoon shift sleep duration×2)+(Night shift sleep duration×2)+(Rest day sleep duration×2)]/8. For workers who do not participate in shift work, sleep duration is calculated as the weighted average of sleep times during a typical work/rest cycle on workdays and non-workdays. In this study, average sleep duration <7 hours/day is defined as "insufficient sleep," 7-8 hours/day as "normal sleep," and >8 hours/day as "adequate sleep."[3]。

**Definition and Classification of Covariates**

1. Marital Status: Due to the small proportion of participants categorized as "divorced," "remarried," or "widowed," all categories other than "unmarried" were combined and defined as "married."

2. Monthly household income: Calculated as the total monthly household income divided by the number of household members.

3. Educational Level: Due to the small proportion of participants in certain educational categories, this study defined "Elementary school and below" as "Primary"; "Junior high school," "High school," and "Vocational school" as "Intermediate"; and "Associate degree," "Bachelor's degree," and "Graduate degree and above" as "Advanced."

4. Smoking Status: Smoking status was assessed based on self-reported information. Smoking is defined as smoking at least one cigarette per day and having done so continuously for more than six months (according to WHO standards). Those not meeting this criterion are defined as "never smokers"; individuals who have quit smoking for six months or more by the date of the survey are classified as "former smokers"; and those meeting the smoking criterion but not the quitting criterion are classified as "current smokers."

5. Drinking status: Drinking habits were assessed based on self-reported data. Participants who reported consuming alcohol at least once per week in the past 12 months were classified as "drinking." Those who did not meet this criterion were classified as "never drinking." Participants who had abstained from alcohol for six months or more by the time of the survey were categorized as "Ever drinking," while those who met the drinking criterion but had not abstained for six months were classified as "Current drinking."

6. Salt taste preference: Dietary salt intake is categorized into mild, moderate, and high. Mild intake is defined as a daily sodium intake of 6 grams or less (2400 mg/day), moderate intake as 6 to 8 grams of sodium per day (2400 to 4000 mg/day), and high intake as 10 grams of sodium or more per day (4000 mg/day).

7. Physical activity level: Physical activity levels in this study were classified into "low," "moderate," and "high" categories according to the International Physical Activity Questionnaire (long form) [4] for calculating and scoring metabolic equivalents.。

8. DASH Score: The Dietary Approaches to Stop Hypertension (DASH) score [5] was calculated based on the types and frequencies of food consumption among coal miners, in accordance with the DASH dietary pattern for hypertension control.

9. Central Obesity: Defined as a waist circumference greater than 85 cm for men and greater than 80 cm for women[6].

10. Hypertension: Defined as a systolic blood pressure of ≥140 mmHg and/or a diastolic blood pressure of ≥90 mmHg. Participants who are currently receiving antihypertensive treatment are also classified as "hypertensive."

11. Abnormal Liver Function: Defined as having at least one of the following criteria: ALT > 40 U/L, AST > 40 U/L, GGT > 60 U/L for men, or GGT > 40 U/L for women[7].

12. Dyslipidemia: Defined as meeting at least one of the following criteria: total cholesterol ≥ 6.22 mmol/L, low-density lipoprotein (LDL) ≥ 4.11 mmol/L, high-density lipoprotein (HDL) ≤ 1.04 mmol/L, triglycerides ≥ 2.32 mmol/L, or currently receiving lipid-lowering treatment[8].

13. Dust exposure: Dust refers to the particulate matter that workers may be exposed to during production processes, including inorganic, organic, or mixed dusts (GBZ/T 229.1-2010). Total dust concentration in the workplace air is measured using filter membranes, with the concentration calculated based on the increase in filter weight and the volume of collected air. When dust concentration is ≤ 50 mg/m³, 37mm or 40mm diameter filters are used; otherwise, 75mm diameter filters are employed.

14. Heat exposure: Exposure to high temperature is defined as an average Wet-Bulb Globe Temperature (WBGT) index ≥ 25°C during production processes (GBZ/T 229.3-2010). The WBGT index is measured using a Wet-Bulb Globe Temperature meter. For each workplace, 4 to 6 measurement points are selected, with the layout of these points determined based on the specific conditions of the workplace. Each point is measured three times, and the final value is the average of these measurements.。

15. CO exposure：Industrial toxicants refer to various harmful chemicals that workers may be exposed to during production processes, with a primary focus on carbon monoxide (CO) in this study (GBZ/T 160.28-2004). CO in the workplace air is pumped into a nondispersive infrared (NDIR) analyzer, which selectively absorbs CO's infrared radiation. The concentration of CO is determined based on the absorption values. The permissible average exposure concentration of CO for an 8-hour workday or a 40-hour workweek is ≤ 20 mg/m³.

16. Noise exposure: In coal mines, workers are exposed to noise environments where the equivalent sound level is ≥80 dB for an 8-hour workday or a 40-hour workweek, which may be harmful to health and hearing (GBZ/T 229.4-2012). Noise levels in the workplace are measured using a sound level meter. If the noise field is uniformly distributed, measurements are taken at three points and averaged. If the noise field is not uniform, the area is divided into several noise zones, with two measurement points selected per zone, and the average is calculated.

**Genetic testing of MTNR1B, BMAL1, and BMAL2**

Primers were designed and synthesized by Shanghai Tianhao Biotechnology Co., Ltd. The upstream and downstream sequences of the primers are listed in Table S9. After diluting the primers, a multiplex polymerase chain reaction (PCR) was performed. Details of the multiplex PCR reaction system are provided in Tables S10 and S11. To a 20 μL PCR product, 5 U of shrimp alkaline phosphatase (SAP) and 2 U of Exonuclease I were added, followed by incubation at 37°C for 1 hour and inactivation at 75°C for 15 minutes. Ligation reaction primers are listed in Table S12. Ligation reaction premix was prepared according to Table S13, and the ligation reaction conditions are detailed in Table S14.

This study, supported by Shanghai Tianhao Biotechnology Co., Ltd., used the iMLDR™ (Improved Multiplex Ligation Detection Reaction) SNP genotyping technology to determine genotypes. The steps are as follows: Target SNP regions are first amplified in a single system using multiplex PCR. The amplified products are purified using Exonuclease I and SAP, then used as templates for the subsequent ligation reaction. In each ligation reaction, each SNP site includes two 5'-end allele-specific probes (with 3' ends being either allele-specific bases or sequences for insertion/deletion polymorphisms) and a fluorescently labeled 3'-end site-specific probe. Ligation products are differentiated by capillary electrophoresis on an ABI3730XL.

As shown in Table S15, theoretical and observed values of each gene locus in the control group were compared. The results indicate that loci rs10830963, rs1387153, rs11022775, rs7950226, rs7958822, and rs11605924 in the study population conform to Hardy-Weinberg equilibrium (P > 0.05), demonstrating good representativeness.

**Establishment of CICRD**

Our study used the KMO test and Bartlett's sphericity test to determine whether the data were suitable for factor analysis. Table S16 shows that the KMO value was 0.774 (>0.500), and Bartlett's sphericity test was significant (P<0.001), indicating that the selected variables were suitable for factor analysis.

Our study developed a comprehensive assessment system for circadian rhythm disorders using seven indicators from shift work, light exposure, and sleep: duration of shift work, cumulative number of night shifts, cumulative duration of night shifts, average frequency of night shifts, nighttime light exposure, insomnia status, and average sleep duration. The associations between these indicators with Type 2 Diabetes Mellitus are detailed in Table S17. By normalizing and adjusting negative indicators, we ensured that all indicators were positively related to the risk of circadian rhythm disorders.

After performing principal component analysis on the seven indicators comprising the CICRD, Table S18 presents the three extracted principal components, and Table S19 shows the total variance explained by them. The rotation results in Table S19 show that the factor loadings for duration of shift work, night shift duration, and frequency are high on F1, labeled as the "Shift Work Factor." Insomnia status and sleep duration have higher factor loadings on F2, which is labeled as the "Sleep Factor." Nighttime light exposure has a higher factor loading on F3, which is labeled as the "Light Exposure Factor.". The scores for the three common factors are calculated as shown in Formulas (1), (2), and (3). By combining the percentage of variance explained by each common factor with its contribution to the cumulative variance, the weight for each common factor was determined. The CICRD score calculation formula is shown in Formula (4). Finally, the normalized values of each circadian rhythm disorder-related indicator were substituted into the formula to obtain the specific CICRD value.

$\begin{matrix} F_{1}=V_{1}\times0.270+V_{2}\times0.290+V_{3}\times0.293+V_{4}\times0.236 \\ +V_{5}\times0.004-V_{6}\times0.036-V_{7}\times0.024 \end{matrix}$ （1）

$\begin{matrix} F_{2}=V_{1}\times(-0.006)-V_{2}\times0.022-V_{3}\times0.032+V_{4}\times0.004 \\ -V_{5}\times0.002+V_{6}\times0.650+V_{7}\times0.0641 \end{matrix}$ （2）

$\begin{matrix} F_{3}=V_{1}\times0.027-V_{2}\times0.002-V_{3}\times0.002-V_{4}\times0.016 \\ +V_{5}\times0.999-V_{6}\times0.027+V_{7}\times0.022 \end{matrix}$ （3）

$F=\frac{48.155\times F_{1}+17.318\times F_{2}+14.298\times F_{3}}{79.771}$ （4）

In the formula:

*F_1_*~*F_3_*: represent the shift work factor, sleep factor, and light exposure factor, respectively

*V_1_*~*V_7_*: represent the duration of shift work, cumulative number of night shifts, cumulative duration of night shifts, average frequency of night shifts, light exposure, insomnia status, and average sleep duration, respectively

*F:* CICRD

**References**

1. Stevens RG, Hansen J, Costa G, Haus E, Kauppinen T, Aronson KJ, et al. Considerations of circadian impact for defining 'shift work' in cancer studies: IARC Working Group Report. Occupational and environmental medicine. 2011;68(2):154-62. doi: 10.1136/oem.2009.053512.

2. Soldatos CR, Dikeos DG, research TJPJJop. Athens Insomnia Scale: validation of an instrument based on ICD-10 criteria. Journal of psychosomatic research 2000;48(6):555-60. doi: 10.1016/s0022-3999(00)00095-7.

3. Wang Y. Association of Occupational Hazard Exposome,Circadian Rhythm-related Genes and TheirInteractions with MetS in Iron and Steel Workers. North China University of Science and Technology. 2019.

4. Craig CL, Marshall AL, Sjöström M, Bauman AE, Booth ML, Ainsworth BE, et al. International physical activity questionnaire: 12-country reliability and validity. Medicine and science in sports and exercise. 2003;35(8):1381-95. doi: 10.1249/01.Mss.0000078924.61453.Fb.

5. Fung TT, Chiuve SE, McCullough ML, Rexrode KM, Logroscino G, medicine FBHJAoi. Adherence to a DASH-style diet and risk of coronary heart disease and stroke in women. Archives of internal medicine. 2008;168(7):713-20. doi: 10.1001/archinte.168.7.713.

6. Reutrakul S, clinical EVCJM, experimental. Sleep influences on obesity, insulin resistance, and risk of type 2 diabetes. Metabolism: clinical and experimental. 2018;84:56-66. doi: 10.1016/j.metabol.2018.02.010.

7. Li Q, Zhang S, Yu M, Wang L, Wang Z, Zhang X, et al. Rotating night shift work and liver enzymes-associated abnormalities among steelworkers: a cross-sectional study from a Chinese cohort. International archives of occupational and environmental health. 2022;95(10):1935-44. doi: 10.1007/s00420-022-01894-0.

8. Jianjun ZJGRZPLGZDL. Guidelines for the prevention and treatment of dyslipidemia in Chinese adults (2016 revision). Chinese Circulation Journal. 2016;31(10):937-53.

**List of content**

**Tables**

**Table S1.** Stratified and interaction analysis of the association between CICRD and T2DM in coal miners

**Table S2.** PCR primer sequences and Tm values

**Table S3.** Multiplex PCR reaction system

**Table S4.** Multiplex PCR reaction conditions

**Table S5.** Primers for ligation reactions

**Table S6.** Composition of the ligation reaction system

**Table S7.** Conditions for ligation reaction

**Table S8.** Hardy-weinberg balance test for each locus in the control group

**Table S9.** KMO and Bartlett sphericity tests

**Table S10.** Logistic regression analysis of circadian rhythm disorders indicators and T2DM in coal miners

**Table S11.** Total variance explained by components of CICRD

**Table S12.** Factor score coefficient matrix for components of CICRD

**Table S13**. Comparison of demographic characteristics between case and control groups

**Table S14.** Association of MTNR1B gene rs10830963, BMAL2 gene rs7958822, and CRY2 gene rs11605924 with T2DM in male coal miners

**Table S15.** Gene-Gene multiplicative and additive Interactions in Type 2 Diabetes risk among coal miners

**Table S16.** Gene-Gene interaction models identified by GMDR

**Table S17.** Interaction of the four-factor combination model

**Table S18.** Interaction effects of the five-factor combination model

**Figures**

**Figure S1**. Relationship between sample size and statistical power

**Figure S2.** Dose-Response relationship between CICRD and T2DM in coal miners

**Figure S3.** Interaction model of four factors determined by GMDR

**Figure S4.** Interaction model of five factors determined by GMDR

**Table S1.** Stratified and interaction analysis of the association between CICRD and T2DM in coal miners

| Group | n, (%) | OR (95% CI) | | | | P _interaction_ |
| --- | --- | --- | --- | --- | --- | --- |
|  |  | <0.1839 | 0.1839~ | 0.2782~ | ≥0.3848 |  |
| Age |  |  |  |  |  | 0.295 |
| 20~ | 592 (14.5) | 1.00 | 0.97 (0.40~2.31) | 1.17 (0.46~3.00) | 1.53 (0.51~4.57) |  |
| 30~ | 1764 (43.4) | 1.00 | 1.27 (0.81~1.99) | 2.06 (1.31~3.24) | 3.66 (2.13~6.30) |  |
| 40~ | 1083 (26.6) | 1.00 | 0.84 (0.47~1.50) | 0.95 (0.56~1.61) | 2.21 (1.31~3.74) |  |
| ≥50 | 631 (15.5) | 1.00 | 1.41 (0.60~3.32) | 2.07 (0.90~4.77) | 2.53 (1.22~5.25) |  |
| Sex |  |  |  |  |  | 0.773 |
| male | 3878 (95.3) | 1.00 | 1.12 (0.83~1.51) | 1.51 (1.13~2.01) | 2.35 (1.79~3.13) |  |
| Female | 192 (4.7) | 1.00 | — | — | — |  |
| Smoking Status |  |  |  |  |  | 0.307 |
| Never smoking | 1607 (39.5) | 1.00 | 0.82 (0.46~1.49) | 1.72 (1.00~2.95) | 2.61 (1.46~4.64) |  |
| Former smoking | 298 (7.3) | 1.00 | 0.42 (0.17~1.02) | 0.93 (0.40~2.13) | 1.13 (0.53~2.42) |  |
| Current smoking | 2165 (53.2) | 1.00 | 1.58 (1.06~2.35) | 1.89 (1.26~2.83) | 3.94 (2.59~5.99) |  |
| Drinking status |  |  |  |  |  | 0.042 |
| Never drinking | 953 (23.4) | 1.00 | 1.01 (0.54~1.91) | 0.51 (0.25~1.06) | 2.15 (1.11~4.15) |  |
| Ever drinking | 192 (4.7) | 1.00 | 4.35 (0.69~27.49) | 6.66 (1.00~44.42) | 8.33 (1.35~51.36) |  |
| Current drinking | 2925 (71.9) | 1.00 | 1.09 (0.77~1.53) | 1.80 (1.29~2.51) | 2.76 (1.94~3.94) |  |
| Dyslipemia |  |  |  |  |  | 0.082 |
| No | 3038 (74.6) | 1.00 | 0.94 (0.67~1.33) | 1.34 (0.97~1.86) | 2.39 (1.72~3.31) |  |
| Yes | 1032 (25.4) | 1.00 | 1.34 (0.78~2.31) | 1.93 (1.16~3.22) | 3.08 (1.84~5.16) |  |
| Abnormal liver function |  |  |  |  |  | 0.639 |
| No | 3268 (80.3) | 1.00 | 0.83 (0.60~1.16) | 1.18 (0.86~1.61) | 2.32 (1.71~3.15) |  |
| Yes | 802 (19.7) | 1.00 | 2.66 (1.39~5.08) | 3.55 (1.90~6.66) | 4.29 (2.24~8.22) |  |
| Abnormal renal function |  |  |  |  |  | 0.105 |
| No | 3869 (95.1) | 1.00 | 1.12 (0.84~1.51) | 1.60 (1.21~2.12) | 2.68 (2.02~3.55) |  |
| Yes | 201 (4.9) | 1.00 | 0.16 (0.03~0.90) | 0.15 (0.03~0.90) | 0.64 (0.15~2.77) |  |
| Hypertension |  |  |  |  |  | 0.450 |
| No | 2627 (64.5) | 1.00 | 1.17 (0.81~1.70) | 1.63 (1.14~2.31) | 2.78 (1.92~4.02) |  |
| Yes | 1443 (35.5) | 1.00 | 0.89 (0.56~1.42) | 1.32 (0.85~2.07) | 2.35 (1.55~3.57) |  |
| Heat exposure |  |  |  |  |  | <0.001 |
| No | 377 (9.3) | 1.00 | 0.23 (0.05~1.06) | 0.07 (0.01~0.74) | 0.49 (0.15~1.53) |  |
| Yes | 3693 (90.7) | 1.00 | 1.29 (0.95~1.75) | 1.90 (1.41~2.55) | 3.19 (2.38~4.29) |  |
| Noise exposure |  |  |  |  |  | 0.917 |
| No | 1878 (46.1) | 1.00 | 0.92 (0.58~1.47) | 1.35 (0.88~2.08) | 2.18 (1.43~3.31) |  |
| Yes | 2192 (53.9) | 1.00 | 1.21 (0.84~1.76) | 1.64 (1.15~2.36) | 3.00 (2.08~4.34) |  |
| Dust exposure |  |  |  |  |  | 0.121 |
| No | 1238 (30.4) | 1.00 | 0.77 (0.47~1.26) | 1.17 (0.73~1.88) | 1.66 (1.02~2.70) |  |
| Yes | 2832 (69.6) | 1.00 | 1.27 (0.88~1.82) | 1.77 (1.26~2.50) | 3.32 (2.36~4.66) |  |
| CO |  |  |  |  |  | 0.004 |
| No | 939 (23.1) | 1.00 | 1.23 (0.69~2.17) | 1.05 (0.58~1.91) | 1.41 (0.83~2.41) |  |
| Yes | 3131 (76.9) | 1.00 | 1.08 (0.77~1.52) | 1.76 (1.27~2.42) | 3.26 (2.35~4.53) |  |

Note: The variables adjusted for in the model include age, sex, marital status, monthly household income per capita, educational level, smoking status, drinking status, salt taste preference, physical activity level, DASH score, abnormal liver function, abnormal renal function, dyslipidemia, hypertension, family history of diabetes, and occupational hazards (CO, noise, dust, and heat); *P* _interaction_: multiplicative interaction.

**Table S2.** PCR primer sequences and Tm values

| Gene | Locus |  | Primer Sequence | Tm (℃) |
| --- | --- | --- | --- | --- |
| MTNR1B | rs10830963 | Upstream | GAATTGGCATTTCTGGGGTACG | 64.69 |
|  |  | Downstream | GGCCACAGTGCAGACTGTTTTC | 64.32 |
|  | rs1387153 | Upstream | GGTAACACATGGAAAATGCTTGGTAAC | 64.60 |
|  |  | Downstream | TATGCAGAACACCTTATCCATTACCC | 63.31 |
| BMAL1 | rs11022775 | Upstream | AGGTGTGCACCTGCCTCTTTTC | 65.02 |
|  |  | Downstream | CCTACCCCGTGTTCACCTGTGT | 65.48 |
|  | rs7950226 | Upstream | GAAGGGGTCTGGGGAATCACTT | 64.77 |
|  |  | Downstream | CTCTGAGGGCCACATTCACAAG | 64.28 |
| BMAL2 | rs7958822 | Upstream | CTGGCCCATCCTTACTGGTCTG | 65.19 |
|  |  | Downstream | AAAGTGAGAGCCCAAGGCAGAG | 64.31 |
| CRY2 | rs11605924 | Upstream | TTCCCAGATGCTGAGAGCTGAA | 64.63 |
|  |  | Downstream | CCCTTTGGGCAAACAGAAACAT | 64.37 |

**Table S3.** Multiplex PCR reaction system

| Reagent | Amount |
| --- | --- |
| 10×PerfectStart^®^ Taq buffer | 2.0μL |
| 10×GC Enhancer | 6.0μL |
| dNTP (2.5mM) | 2.4μL |
| MgCl_2_ (25m) | 0.8μL |
| Probe Mix | 2.0μL |
| Hot Star Taq (2.5U/μL) | 0.4μL |
| ddH_2_O | 5.4μL |
| Total Volume | 19.0μL |

**Table S4.** Multiplex PCR reaction conditions

| Steps | Denaturation | Annealing | Annealing | Hold | Number of Cycles |
| --- | --- | --- | --- | --- | --- |
| First step | 95ºC 2min | — | — | — | 1 |
| Second step | 94ºC 20s | 65ºC 40s (-0.5ºC/cycle) | 72ºC 1.5min | — | 11 |
| Third step | 94ºC 20s | 59ºC 30s | 72ºC 1.5min | — | 24 |
| Fourth step | — | — | 72ºC 2.0min | 4ºC | 1 |

**Table S5.** Primers for ligation reactions

| SNP | Primer Name | Seq (5’-3’) |
| --- | --- | --- |
| rs10830963 | rs10830963_CR | TGTTCGTGGGCCGGATTAGTCCAGGCAGTTACTGGTTCTGGATTGG |
|  | rs10830963_GR | TGTTCGTGGGCCGGATTAGTTTCCAGGCAGTTACTGGTTCTGGATTGC |
|  | rs10830963_RP | AGATGGTGTGAATTCTTAGCATCACTGT |
| rs11022775 | rs11022775_CR | TGTTCGTGGGCCGGATTAGTccctctctcctcaaacttccaAcg |
|  | rs11022775_TR | TGTTCGTGGGCCGGATTAGTTTccctctctcctcaaacttccaGcA |
|  | rs11022775_RP | tctccttagtctccattctccgcTT |
| rs11605924 | rs11605924_AR | TTCCGCGTTCGGACTGATATGCAGCAGAACAGTAAAGTGCCTCGCT |
|  | rs11605924_CR | TTCCGCGTTCGGACTGATATTTGCAGCAGAACAGTAAAGTGCCTCACG |
|  | rs11605924_RP | CAGGTACCAATGTGAATAGCTCAGAGA |
| r1387153 | rs1387153_CR | TGTTCGTGGGCCGGATTAGTgaatgctagcaaattaacttactgcccAgg |
|  | rs1387153_TR | TGTTCGTGGGCCGGATTAGTTTgaatgctagcaaattaacttactgcccGgA |
|  | rs1387153_RP | aactcaattttcttatctgtaaaaGCAACACA |
| rs7950226 | rs7950226_GR | TTCCGCGTTCGGACTGATATCACATTCACAAGGGAAATATAGAAACATGTAGGAAC |
|  | rs7950226_AR | TTCCGCGTTCGGACTGATATTTCACATTCACAAGGGAAATATAGAAACATGTAGGGAT |
|  | rs7950226_RP | ACACATGCGTGTATGTAGAYACATTTTTAAAG |
| rs7958822 | rs7958822_GF | TTCCGCGTTCGGACTGATATCCATTCTAACCATTCATATCTCTTCCCACG |
|  | rs7958822_AF | TTCCGCGTTCGGACTGATATTTCCATTCTAACCATTCATATCTCTTCCCACA |
|  | rs7958822_FP | TGTCAACCTTTTACTTTCCCCTATAACTC |

**Table S6.** Composition of the ligation reaction system

| Reagent | Volume |
| --- | --- |
| 10x ligase buffer | 1.00μL |
| Labelp Mix | 0.25μL |
| Ligase Primer Mix (1uM) | 0.40μL |
| DNA ligase (50U/μL) | 1.00μL |
| ddH_2_O | 3.35μL |
| Total Volume | 6.00μL |

**Table S7.** Conditions for ligation reaction

| Steps | Denaturation | Ligation | Hold | Number of Cycles |
| --- | --- | --- | --- | --- |
| First step | 94ºC 1.0min | 56ºC 4.0min | — | 38 |
| Second step | — | — | 4ºC | — |

**Table S8.** Hardy-weinberg balance test for each locus in the control group

| Genes | SNPs | Genotype | Observed value | Theoretical value | *χ^2^* | *P* |
| --- | --- | --- | --- | --- | --- | --- |
| MTNR1B | rs10830963 | CC | 191 | 179.38 | 2.681 | 0.262 |
|  |  | CG | 190 | 213.25 |  |  |
|  |  | GG | 75 | 63.38 |  |  |
|  | rs1387153 | CC | 181 | 175.01 | 0.704 | 0.703 |
|  |  | CT | 203 | 214.97 |  |  |
|  |  | TT | 72 | 66.01 |  |  |
| BMAL1 | rs11022775 | CC | 373 | 372.25 | 0.076 | 0.963 |
|  |  | CT | 78 | 79.51 |  |  |
|  |  | TT | 5 | 4.25 |  |  |
|  | rs7950226 | AA | 151 | 157.51 | 0.795 | 0.672 |
|  |  | GA | 234 | 220.98 |  |  |
|  |  | GG | 71 | 77.51 |  |  |
| BMAL2 | rs7958822 | AA | 24 | 24.64 | 0.014 | 0.993 |
|  |  | GA | 164 | 162.72 |  |  |
|  |  | GG | 268 | 268.64 |  |  |
| CRY2 | rs11605924 | AA | 270 | 270.18 | 0.001 | 0.999 |
|  |  | AC | 162 | 161.64 |  |  |
|  |  | CC | 24 | 24.18 |  |  |

**Table S9.** KMO and Bartlett sphericity tests

| Inspection item |  | Numerical value |
| --- | --- | --- |
| KMO sample appropriateness measure | — | 0.774 |
| Bartlett sphericity test | Approximate chi-square | 23413.475 |
|  | Degree of freedom | 21 |
|  | *P* | <0.001 |

**Table S10.** Logistic regression analysis of circadian rhythm disorders indicators and T2DM in coal miners

| Variables |  | n, (%) | *OR* (95% *CI*) | | |
| --- | --- | --- | --- | --- | --- |
|  |  |  | Model 1 | Model 2 | Model 3 |
| duration of shift work (year) | <0.37 | 1017 (24.99) | 1.00 | 1.00 | 1.00 |
|  | 0.37~ | 1010 (24.81) | 1.70 (1.31~2.20) | 1.92 (1.40~2.59) | 1.82 (1.34~2.47) |
|  | 9.34~ | 1013 (24.89) | 1.55 (1.20~1.99) | 1.74 (1.30~2.35) | 1.61 (1.19~2.18) |
|  | ≥15.59 | 1030 (25.31) | 1.71 (1.33~2.20) | 1.95 (1.46~2.62) | 1.93 (1.43~2.60) |
|  | Trend test | — | 1.17 (1.08~1.26) | 1.22 (1.12~1.34) | 1.21 (1.10~1.33) |
|  | Per *SD* increase | — | 1.15 (1.06~1.26) | 1.18 (1.06~1.31) | 1.18 (1.06~1.31) |
| cumulative number of night shifts | <21 | 1017 (24.99) | 1.00 | 1.00 | 1.00 |
|  | 21~ | 1010 (24.82) | 1.39 (1.06~1.81) | 1.71 (1.25~2.32) | 1.63 (1.19~2.23) |
|  | 494~ | 1025 (25.18) | 1.50 (1.17~1.93) | 1.67 (1.24~2.25) | 1.52 (1.12~2.05) |
|  | ≥1171 | 1018 (25.01) | 2.12 (1.65~2.73) | 2.29 (1.71~3.07) | 2.25 (1.67~3.02) |
|  | Trend test | — | 1.27 (1.17~1.37) | 1.29 (1.17~1.42) | 1.28 (1.16~1.40) |
|  | Per *SD* increase | — | 1.23 (1.13~1.34) | 1.25 (1.13~1.38) | 1.26 (1.14~1.39) |
| cumulative duration of night shifts (h) | <131 | 1017 (24.99) | 1.00 | 1.00 | 1.00 |
|  | 131~ | 1018 (25.01) | 1.46 (1.12~1.90) | 1.78 (1.31~2.41) | 1.71 (1.26~2.33) |
|  | 3621~ | 1018 (25.01) | 1.38 (1.07~1.78) | 1.53 (1.13~2.06) | 1.37 (1.01~1.86) |
|  | ≥8643 | 1017 (24.99) | 2.20 (1.71~2.82) | 2.33 (1.74~3.12) | 2.33 (1.73~3.13) |
|  | Trend test | — | 1.26 (1.17~1.37) | 1.29 (1.18~1.42) | 1.28 (1.17~1.41) |
|  | Per *SD* increase | — | 1.23 (1.13~1.33) | 1.23 (1.12~1.35) | 1.24 (1.13~1.37) |
| average frequency of night shifts (nights/month) | <3.04 | 1011 (24.84) | 1.00 | 1.00 | 1.00 |
|  | 3.04~ | 1364 (33.51) | 1.24 (0.97~1.59) | 1.55 (1.17~2.06) | 1.46 (1.09~1.94) |
|  | 3.80~ | 556 (13.66) | 1.48 (1.10~2.00) | 1.64 (1.16~2.32) | 1.54 (1.08~2.18) |
|  | ≥7.60 | 1139 (27.99) | 2.45 (1.93~3.11) | 2.53 (1.92~3.34) | 2.51 (1.89~3.32) |
|  | Trend test | — | 1.36 (1.26~1.46) | 1.34 (1.23~1.46) | 1.34 (1.23~1.46) |
|  | Per *SD* increase | — | 1.12 (1.09~1.15) | 1.13 (1.09~1.16) | 1.12 (1.09~1.16) |
| nighttime light exposure | Darkest | 1259 (30.93) | 1.00 | 1.00 | 1.00 |
|  | Moderate | 2047 (50.30) | 1.28 (1.05~1.56) | 1.42 (1.13~1.79) | 1.43 (1.14~1.81) |
|  | Brightest | 764 (18.77) | 1.54 (1.21~1.96) | 1.63 (1.23~2.16) | 1.64 (1.23~2.19) |
|  | Trend test | — | 1.24 (1.10~1.40) | 1.29 (1.12~1.48) | 1.30 (1.13~1.50) |
| average sleep duration (h/d) | <7 | 1036 (25.45) | 1.00 | 1.00 | 1.00 |
|  | 7~8 | 2334 (57.35) | 0.74 (0.61~0.90) | 0.73 (0.59~0.92) | 0.72 (0.58~0.90) |
|  | 8~ | 700 (17.20) | 0.70 (0.54~0.91) | 0.72 (0.53~0.96) | 0.68 (0.50~0.91) |
|  | Trend test | — | 0.82 (0.72~0.93) | 0.83 (0.71~0.96) | 0.81 (0.70~0.94) |
|  | Per *SD* increase | — | 0.89 (0.82~0.97) | 1.06 (0.85~1.32) | 1.09 (0.88~1.37) |
| insomnia status | No sleep disorder | 2776 (68.20) | 1.00 | 1.00 | 1.00 |
|  | Suspected insomnia | 861 (21.15) | 1.12 (0.91~1.37) | 1.11 (0.89~1.39) | 1.00 (0.78~1.28) |
|  | Insomnia | 433 (10.65) | 1.85 (1.45~2.36) | 1.78 (1.37~2.32) | 1.62 (1.21~2.16) |
|  | Trend test | — | 1.30 (1.16~1.46) | 1.21 (1.05~1.38) | 1.21 (1.05~1.38) |

Model 1: adjusted for age and gender;

Model 2: further adjusted for marital status, family income per capita, education level, smoking status, drinking status, salt taste preference, physical activity level, DASH score, abnormal liver function, abnormal renal function, dyslipidemia, hypertension, and family history of diabetes;

Model 3: further adjusted for occupational hazards (CO, noise, dust and heat);

SD: standard deviation

**Table S11.** Total variance explained by components of CICRD

| Component | Initial | | | After rotation | | |
| --- | --- | --- | --- | --- | --- | --- |
|  | Eigenvalue | Variance Percentage (%) | Cumulative Percentage (%) | Eigenvalue | Variance Percentage (%) | Cumulative Percentage (%) |
| 1 | 3.394 | 48.482 | 48.482 | 3.371 | 48.155 | 48.155 |
| 2 | 1.190 | 16.998 | 65.480 | 1.212 | 17.318 | 65.473 |
| 3 | 1.000 | 14.290 | 79.771 | 1.001 | 14.298 | 79.771 |
| 4 | 0.792 | 11.311 | 91.081 | — | — | — |
| 5 | 0.470 | 6.720 | 97.801 | — | — | — |
| 6 | 0.139 | 1.984 | 99.785 | — | — | — |
| 7 | 0.015 | 0.215 | 100 | — | — | — |

**Table S12.** Factor score coefficient matrix for components of CICRD

| Component | ID | Principal Factors | | | Rotated Component Matrix | | |
| --- | --- | --- | --- | --- | --- | --- | --- |
|  |  | *F*_1_ | *F*_2_ | *F*_3_ | *F*_1_ | *F*_2_ | *F*_3_ |
| duration of shift work (year) | *V*_1_ | 0.270 | -0.006 | 0.027 | 0.907 | 0.053 | 0.021 |
| cumulative number of night shifts (night) | *V*_2_ | 0.290 | -0.022 | -0.002 | 0.972 | 0.038 | -0.009 |
| cumulative duration of night shifts (h) | *V*_3_ | 0.293 | -0.032 | -0.002 | 0.981 | 0.027 | -0.008 |
| average frequency of night shifts (nights/month) | *V*_4_ | 0.236 | 0.004 | -0.016 | 0.797 | 0.057 | -0.021 |
| nighttime light exposure | *V*_5_ | 0.004 | -0.002 | 0.999 | -0.009 | 0.004 | 0.999 |
| insomnia status | *V*_6_ | -0.036 | 0.650 | -0.027 | 0.024 | 0.780 | -0.023 |
| average sleep duration (h/d) | *V*_7_ | -0.024 | 0.641 | 0.022 | 0.060 | 0.771 | 0.026 |

**Table S13**. Comparison of demographic characteristics between case and control groups

| Variable | Total population (n=888) | Case Group (n=424) | Control Group (n=464) | *P* |
| --- | --- | --- | --- | --- |
| Age (Year) | 40.23±8.61 | 41.03±8.65 | 39.46±8.50 | 0.008a |
| DASH score | 23.28±2.61 | 23.18±2.51 | 23.37±2.70 | 0.258a |
| CICRD | 0.31±0.15 | 0.33±0.15 | 0.29±0.14 | <0.001a |
| Marital status, n (%) |  |  |  | 0.135 |
| Unmarried | 31 (3.5) | 11 (2.5) | 20 (4.4) |  |
| Married | 857 (96.5) | 421 (97.5) | 436 (95.6) |  |
| Monthly household income (Yuan/Person), n (%) |  |  |  | <0.003b |
| <1509 | 238 (26.8) | 106 (24.5) | 132 (28.9) |  |
| 1509~ | 232 (26.1) | 127 (29.4) | 105 (23.1) |  |
| 1887~ | 198 (22.3) | 79 (18.3) | 119 (26.1) |  |
| 2516~ | 220 (24.8) | 120 (27.8) | 100 (21.9) |  |
| Education level, n (%) |  |  |  | 0.060b |
| Primary level | 15 (1.7) | 11 (2.5) | 4 (0.9) |  |
| Intermediate level | 645 (72.6) | 320 (74.1) | 325 (71.2) |  |
| Advanced level | 228 (25.7) | 101 (23.4) | 127 (27.9) |  |
| Smoking status, n (%) |  |  |  | <0.001 |
| Never smoking | 308 (34.7) | 119 (27.5) | 189 (41.4) |  |
| Ever smoking | 93 (10.5) | 58 (13.4) | 35 (7.7) |  |
| Current smoking | 487 (54.8) | 255 (59.1) | 232 (50.9) |  |
| Drinking status, n (%) |  |  |  | 0.160 |
| Never drinking | 160 (18.0) | 67 (15.5) | 93 (20.4) |  |
| Ever drinking | 54 (6.1) | 28 (6.5) | 26 (5.7) |  |
| Current drinking | 674 (75.9) | 337 (78.0) | 337 (73.9) |  |
| Salt taste preference, n (%) |  |  |  | 0.125b |
| Light | 143 (16.1) | 64 (14.8) | 79 (17.3) |  |
| Moderate | 423 (47.6) | 197 (45.6) | 226 (49.6) |  |
| Salty | 322 (36.3) | 171 (39.6) | 151 (33.1) |  |
| Physical activity level, n (%) |  |  |  | 0.148b |
| Low | 94 (10.6) | 44 (10.2) | 50 (11.0) |  |
| Moderate | 398 (44.8) | 208 (48.1) | 190 (41.7) |  |
| High | 396 (44.6) | 180 (41.7) | 216 (47.3) |  |
| Dyslipidemia, n (%) |  |  |  | 0.003 |
| No | 641 (72.2) | 292 (67.6) | 349 (76.5) |  |
| Yes | 247 (27.8) | 140 (32.4) | 107 (23.5) |  |
| Abnormal liver function, n (%) |  |  |  | 0.003 |
| No | 688 (77.5) | 316 (73.1) | 372 (81.6) |  |
| Yes | 200 (22.5) | 116 (26.9) | 84 (18.4) |  |
| Abnormal Kidney Function, n (%) |  |  |  | 0.404 |
| No | 850 (95.7) | 411 (95.1) | 439 (96.3) |  |
| Yes | 38 (4.3) | 21 (4.9) | 17 (3.7) |  |
| Hypertension, n (%) |  |  |  | 0.001 |
| No | 531 (59.8) | 235 (54.4) | 296 (64.9) |  |
| Yes | 357 (40.2) | 197 (45.6) | 160 (35.1) |  |
| Central Obesity, n (%) |  |  |  | <0.001 |
| No | 616 (69.4) | 266 (61.6) | 350 (76.8) |  |
| Yes | 272 (30.6) | 166 (38.4) | 106 (23.2) |  |
| Family History of Diabetes, n (%) |  |  |  | <0.001 |
| No | 710 (80.0) | 296 (68.5) | 414 (90.8) |  |
| Yes | 178 (20.0) | 136 (31.5) | 42 (9.2) |  |
| Heat exposure, n (%) |  |  |  | 0.069 |
| No | 66 (7.4) | 25 (5.8) | 41 (9.0) |  |
| Yes | 822 (92.6) | 407 (94.2) | 415 (91.0) |  |
| Noise exposure, n (%) |  |  |  | <0.001 |
| No | 409 (46.1) | 172 (39.8) | 237 (52.0) |  |
| Yes | 479 (53.9) | 260 (60.2) | 219 (48.0) |  |
| Dust exposure, n (%) |  |  |  | 0.107 |
| No | 282 (31.8) | 126 (29.2) | 156 (34.2) |  |
| Yes | 606 (68.2) | 306 (70.8) | 300 (65.8) |  |
| CO exposure , n (%) |  |  |  | 0.356 |
| No | 212 (23.9) | 109 (25.2) | 105 (22.6) |  |
| Yes | 676 (76.1) | 323 (74.8) | 353 (77.4) |  |

Note: ^a^ Indicates that the conditions for parametric testing were not met, and the Mann-Whitney U test was used; ^b^ Results were obtained using the Cochran-Armitage trend test; Continuous variables are presented as mean ± SD.

**Table S14.** Association of MTNR1B gene rs10830963, BMAL2 gene rs7958822, and CRY2 gene rs11605924 with T2DM in male coal miners

| SNPs | Model | Genotype | Case Group [n (%)] | Control Group [n (%)] | OR (95% CI) | AIC | BIC |
| --- | --- | --- | --- | --- | --- | --- | --- |
| rs10830963 | Codominance | CC | 191 (41.9) | 140 (32.4) | 1.00 | 1227.7 | 1242.0 |
|  |  | CG | 190 (41.7) | 214 (49.5) | 1.54 (1.15~2.06) |  |  |
|  |  | GG | 75 (16.4) | 78 (18.1) | 1.42 (0.97~2.08) |  |  |
|  | Dominance | CC | 191 (41.9) | 140 (32.4) | 1.00 | 1225.8 | 1235.4 |
|  |  | CG+GG | 265 (58.1) | 292 (67.6) | 1.50 (1.14~1.98) |  |  |
|  | Recessive | CC+CG | 381 (83.5) | 354 (81.9) | 1.00 | 1234.0 | 1243.6 |
|  |  | GG | 75 (16.5) | 78 (18.1) | 1.12 (0.79~1.59) |  |  |
|  | Overdominance | CC+GG | 266 (58.3) | 218 (50.5) | 1.00 | 1228.8 | 1238.4 |
|  |  | CG | 190 (41.7) | 214 (49.5) | 1.37 (1.05~1.79) |  |  |
|  | Additivity | ––– | ––– | ––– | 1.25 (1.03~1.50) | 1229.0 | 1238.5 |
| rs7958822 | Codominance | GG | 268 (58.8) | 216 (50.0) | 1.00 | 1229.3 | 1243.6 |
|  |  | GA | 164 (36.0) | 185 (42.8) | 1.40 (1.06~1.84) |  |  |
|  |  | AA | 24 (5.2) | 31 (7.2) | 1.60 (0.91~2.81) |  |  |
|  | Dominance | GG | 268 (58.8) | 216 (50.0) | 1.00 | 1227.5 | 1237.1 |
|  |  | GA+AA | 188 (41.2) | 216 (50.0) | 1.43 (1.09~1.86) |  |  |
|  | Recessive | GG+GA | 432 (94.7) | 401 (92.8) | 1.00 | 1233.0 | 1242.6 |
|  |  | AA | 24 (5.3) | 31 (7.2) | 1.39 (0.80~2.41) |  |  |
|  | Overdominance | GG+AA | 292 (64.0) | 247 (57.2) | 1.00 | 1230.0 | 1239.6 |
|  |  | GA | 164 (36.0) | 185 (42.8) | 1.33 (1.02~1.75) |  |  |
|  | Additivity | ––– | ––– | ––– | 1.33 (1.07~1.66) | 1227.6 | 1237.2 |
| rs11605924 | Codominance | AA | 270 (59.2) | 224 (51.9) | 1.00 | 1231.4 | 1245.8 |
|  |  | AC | 162 (35.5) | 183 (42.3) | 1.36 (1.03~1.79) |  |  |
|  |  | CC | 24 (5.3) | 25 (5.8) | 1.26 (0.70~2.26) |  |  |
|  | Dominance | AA | 270 (59.2) | 224 (51.9) | 1.00 | 1229.5 | 1239.1 |
|  |  | AC+CC | 186 (40.8) | 208 (48.1) | 1.35 (1.03~1.76) |  |  |
|  | Recessive | AA+AC | 432 (94.7) | 407 (94.2) | 1.00 | 1234.3 | 1243.8 |
|  |  | CC | 24 (5.3) | 25 (5.8) | 1.11 (0.62~1.97) |  |  |
|  | Overdominance | AA+CC | 294 (64.5) | 249 (57.6) | 1.00 | 1230.0 | 1239.6 |
|  |  | AC | 162 (35.5) | 183 (42.4) | 1.33 (1.02~1.75) |  |  |
|  | Additivity | ––– | ––– | ––– | 1.25 (1.01~1.55) | 1230.6 | 1240.1 |
| rs1387153 | Codominance | CC | 181 (39.7) | 159 (36.8) | 1.00 | 1235.5 | 1249.8 |
|  |  | CT | 203 (44.5) | 198 (45.8) | 1.11 (0.83~1.48) |  |  |
|  |  | TT | 72 (15.8) | 75 (17.4) | 1.19 (0.81~1.75) |  |  |
|  | Dominance | CC | 181 (39.7) | 159 (36.8) | 1.00 | 1233.6 | 1243.2 |
|  |  | CT+TT | 275 (60.3) | 273 (63.2) | 1.13 (0.86~1.48) |  |  |
|  | Recessive | CC+CT | 384 (84.2) | 357 (82.6) | 1.00 | 1234.0 | 1243.6 |
|  |  | TT | 72 (15.8) | 75 (17.4) | 1.12 (0.79~1.60) |  |  |
|  | Overdominance | CC+TT | 253 (55.5) | 234 (54.2) | 1.00 | 1234.2 | 1243.8 |
|  |  | CT | 203 (44.5) | 198 (45.8) | 1.05 (0.81~1.37) |  |  |
|  | Additivity | ––– | ––– | ––– | 1.09 (0.91~1.32) | 1233.5 | 1243.1 |
| rs11022775 | Codominance | CC | 373 (81.8) | 342 (79.2) | 1.00 | 1235.2 | 1249.6 |
|  |  | CT | 78 (17.1) | 86 (19.9) | 1.20 (0.86~1.69) |  |  |
|  |  | TT | 5 (1.1) | 4 (0.9) | 0.87 (0.23~3.28) |  |  |
|  | Dominance | CC | 373 (81.8) | 342 (79.2) | 1.00 | 1233.4 | 1243.0 |
|  |  | CT+TT | 83 (18.2) | 90 (20.8) | 1.18 (0.85~1.65) |  |  |
|  | Recessive | CC+CT | 451 (98.9) | 428 (99.1) | 1.00 | 1234.3 | 1243.9 |
|  |  | TT | 5 (1.1) | 4 (0.9) | 0.84 (0.22~3.16) |  |  |
|  | Overdominance | CC+TT | 378 (82.9) | 346 (80.1) | 1.00 | 1233.2 | 1242.8 |
|  |  | CT | 78 (17.1) | 86 (19.9) | 1.20 (0.86~1.69) |  |  |
|  | Additivity | ––– | ––– | ––– | 1.14 (0.84~1.56) | 1233.6 | 1243.2 |
| rs7950226 | Codominance | AA | 151 (33.1) | 158 (36.6) | 1.00 | 1232.6 | 1247.0 |
|  |  | GA | 234 (51.3) | 194 (44.9) | 0.79 (0.59~1.06) |  |  |
|  |  | GG | 71 (15.6) | 80 (18.5) | 1.08 (0.73~1.59) |  |  |
|  | Dominance | AA | 151 (33.1) | 158 (36.6) | 1.00 | 1233.2 | 1242.8 |
|  |  | GA+GG | 305 (66.9) | 274 (63.4) | 0.86 (0.65~1.13) |  |  |
|  | Recessive | AA+GA | 385 (84.4) | 352 (81.5) | 1.00 | 1233.0 | 1242.6 |
|  |  | GG | 71 (15.6) | 80 (18.5) | 1.23 (0.87~1.75) |  |  |
|  | Overdominance | AA+GG | 222 (48.7) | 238 (55.1) | 1.00 | 1230.7 | 1240.3 |
|  |  | GA | 234 (51.3) | 194 (44.9) | 0.77 (0.59~1.01) |  |  |
|  | Additivity | ––– | ––– | ––– | 0.99 (0.82~1.20) | 1234.4 | 1243.9 |

Note: Adjusted for age, marital status, monthly household income, education level, smoking status, drinking status, salt taste preference, physical activity level, DASH score, liver function abnormalities, kidney function abnormalities, dyslipidemia, hypertension, family history of diabetes, CICRD, and occupational hazards (CO, noise, dust, and heat).

**Table S15.** Gene-Gene multiplicative and additive Interactions in Type 2 Diabetes risk among coal miners

| SNP 1 | SNP 2 | Cases | Controls | *OR* (95% CI) | | P _Multiplicative Interaction_ | |
| --- | --- | --- | --- | --- | --- | --- | --- |
|  |  |  |  | Model 1 | Model 2 | Model 1 | Model 2 |
| rs10830963 | rs7958822 |  |  |  |  | 0.298 | 0.248 |
| CC | GG | 69 | 119 | 1.00 | 1.00 |  |  |
|  | GA+AA | 71 | 72 | 1.71 (1.09~2.65) | 2.03 (1.24~3.32) |  |  |
| CG+GG | GG | 147 | 149 | 1.70 (1.17~2.47) | 1.91 (1.26~2.90) |  |  |
|  | GA+AA | 145 | 116 | 2.16 (1.47~3.17) | 2.71 (1.77~4.15) |  |  |
| Trend Test | |  |  | 1.26 (1.12~1.42) | 1.34 (1.17~1.54) |  |  |
| RERI | |  |  | -0.25(-1.18~0.68) | -0.24 (-1.44~0.97) |  |  |
| AP | |  |  | -0.11(-0.55~0.32) | -0.09 (-0.54~0.36) |  |  |
| rs10830963 | rs11605924 |  |  |  |  | 0.777 | 0.667 |
| CC | AA | 70 | 108 | 1.00 | 1.00 |  |  |
|  | AC+CC | 70 | 83 | 1.30 (0.84~2.02) | 1.69 (1.03~2.76) |  |  |
| CG+GG | AA | 154 | 162 | 1.47 (1.01~2.13) | 1.80 (1.18~2.73) |  |  |
|  | AC+CC | 138 | 103 | 2.07 (1.39~3.07) | 2.64 (1.70~4.11) |  |  |
| Trend Test | |  |  | 1.26 (1.12~1.43) | 1.35 (1.18~1.55) |  |  |
| RERI | |  |  | 0.97 (0.17~1.78) | 0.21 (0.11~0.31) |  |  |
| AP | |  |  | 0.47 (0.19~0.75) | 0.46 (0.16~0.75) |  |  |
| rs7958822 | rs11605924 |  |  |  |  | 0.061 | 0.008 |
| GG | AA | 116 | 149 | 1.00 | 1.00 |  |  |
|  | AC+CC | 100 | 119 | 1.08 (0.75~1.55) | 1.06 (0.71~1.59) |  |  |
| GA+AA | AA | 108 | 121 | 1.15 (0.80~1.64) | 1.16 (0.78~1.72) |  |  |
|  | AC+CC | 108 | 67 | 2.07 (1.40~3.06) | 2.77 (1.80~4.27) |  |  |
| Trend Test | |  |  | 1.23 (1.09~1.39) | 1.33 (1.17~1.52) |  |  |
| RERI | |  |  | 0.85 (0.08~1.61) | 1.55 (0.51~2.60) |  |  |
| AP | |  |  | 0.41 (0.11~0.70) | 0.56 (0.32~0.80) |  |  |

Note: Adjusted for age, marital status, per capita monthly household income, education level, smoking status, drinking status, salt taste preference, physical activity level, DASH score, liver function abnormalities, kidney function abnormalities, dyslipidemia, hypertension, family history of diabetes, CICRD, and occupational hazards (CO, noise, dust, and heat)

**Table S16.** Gene-Gene interaction models identified by GMDR

| Model | Training set accuracy | Validation set accuracy | P | Cross-validation consistency |
| --- | --- | --- | --- | --- |
| rs10830963 | 0.5607 | 0.5189 | 9 (0.0730) | 8/12 |
| rs7958822- rs11605924 | 0.5780 | 0.5413 | 10 (0.0193) | 10/12 |
| rs10830963- rs7958822- rs11605924 | 0.6066 | 0.5444 | 9 (0.0730) | 10/12 |
| rs10830963- rs1387153- rs7958822- rs11605924 | 0.6351 | 0.5804 | 11 (0.0032) | 11/12 |
| rs10830963- rs1387153- rs79588226- rs7958822- rs11605924 | 0.6683 | 0.5157 | 8 (0.1938) | 12/12 |
| rs10830963- rs1387153- rs11022775- rs79588226- rs7958822- rs11605924 | 0.6954 | 0.5305 | 9 (0.0730) | 12/12 |

**Table S17.** Interaction of the four-factor combination model

| Model | Dataset | *χ*^2^ | *P* | *OR* (95% *CI*) |
| --- | --- | --- | --- | --- |
| Four-Factor Combination Model | Training Set | 25.427 | <0.001 | 3.13 (2.00~4.91) |
|  | Validation Set | 1.040 | 0.308 | 2.14 (0.48~9.62) |
|  | Entire Dataset | 27.231 | <0.001 | 3.10 (2.01~4.77) |

Note: Adjusted for age, marital status, per capita monthly household income, education level, smoking status, drinking status, salt taste preference, physical activity level, DASH score, liver function abnormalities, kidney function abnormalities, dyslipidemia, hypertension, family history of diabetes, CICRD, and occupational hazards (CO, noise, dust, and heat). Cross-Validation: Refers to randomly dividing the data into 12 parts, using one part as the validation set and the remaining 11 parts as the training set, followed by training and validating the model to ensure balanced testing.

**Table S18.** Interaction effects of the five-factor combination model

| Model | Dataset | *χ*^2^ | *P* | *OR* (95% *CI*) |
| --- | --- | --- | --- | --- |
| Five-Factor Combination Model | Training Set | 90.996 | <0.001 | 7.97 (5.10~12.47) |
|  | Validation Set | 0.581 | 0.446 | 1.51 (0.35~6.45) |
|  | Entire Dataset | 93.944 | <0.001 | 7.38 (4.84~11.25) |

Note: Adjusted for age, marital status, per capita monthly household income, education level, smoking status, drinking status, salt taste preference, physical activity level, DASH score, liver function abnormalities, kidney function abnormalities, dyslipidemia, hypertension, family history of diabetes, and occupational hazards (CO, noise, dust, and heat).


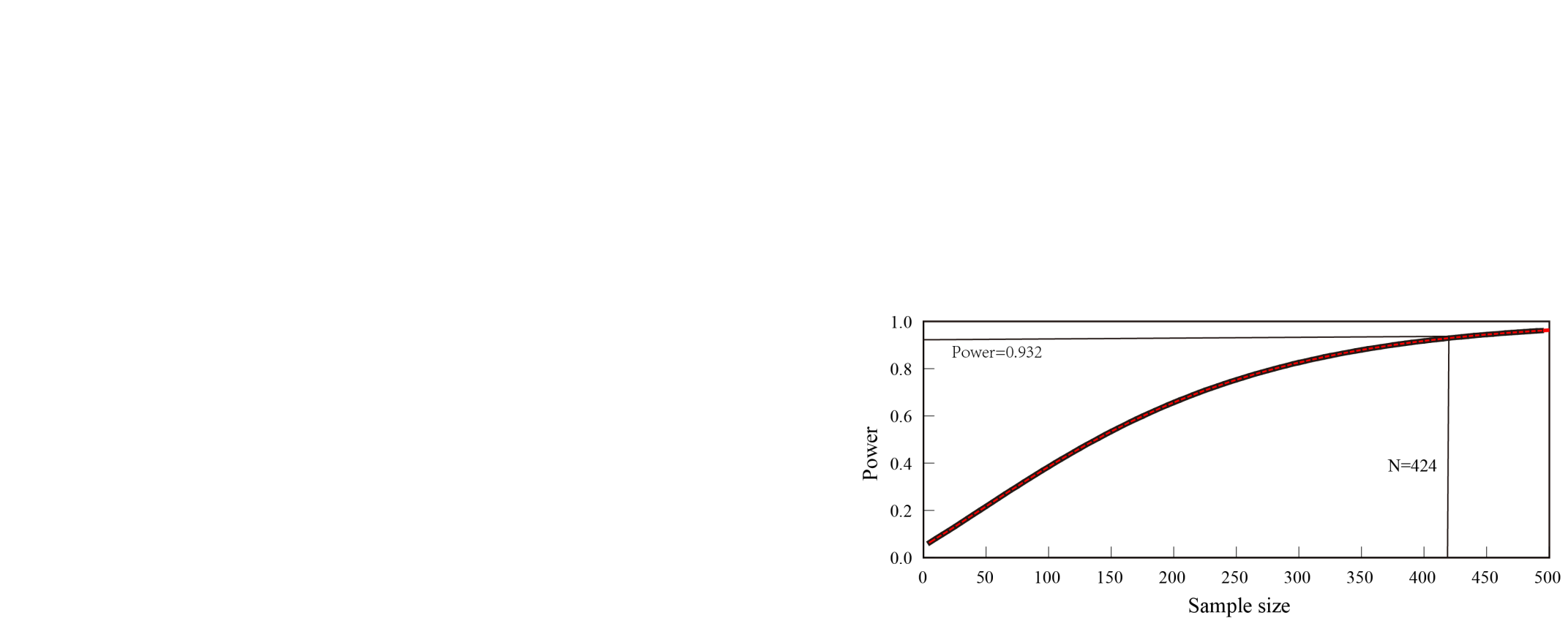


Figure S1. Relationship between sample size and statistical power


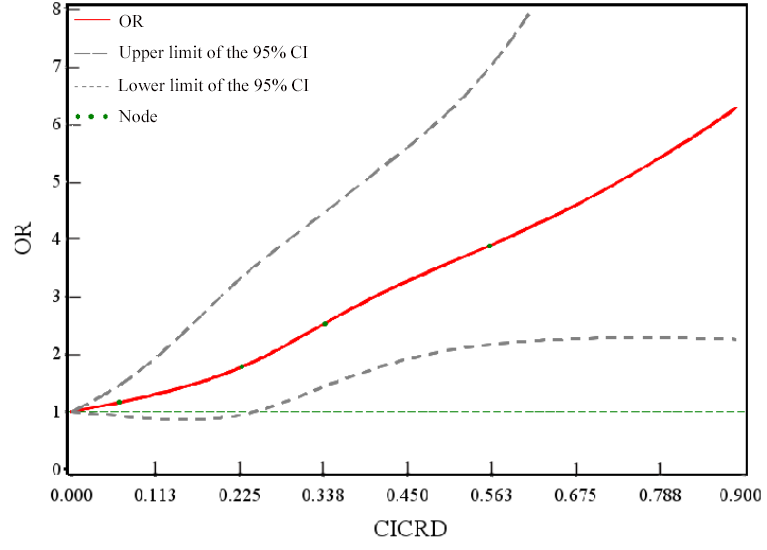


Note: Adjusted for age, marital status, per capita monthly household income, education level, smoking status, drinking status, salt taste preference, physical activity level, DASH score, liver function abnormalities, kidney function abnormalities, dyslipidemia, hypertension, family history of diabetes, and occupational hazards (CO, noise, dust, and heat)

Figure S2. Dose-Response relationship between CICRD and type 2 diabetes mellitus in coal miners


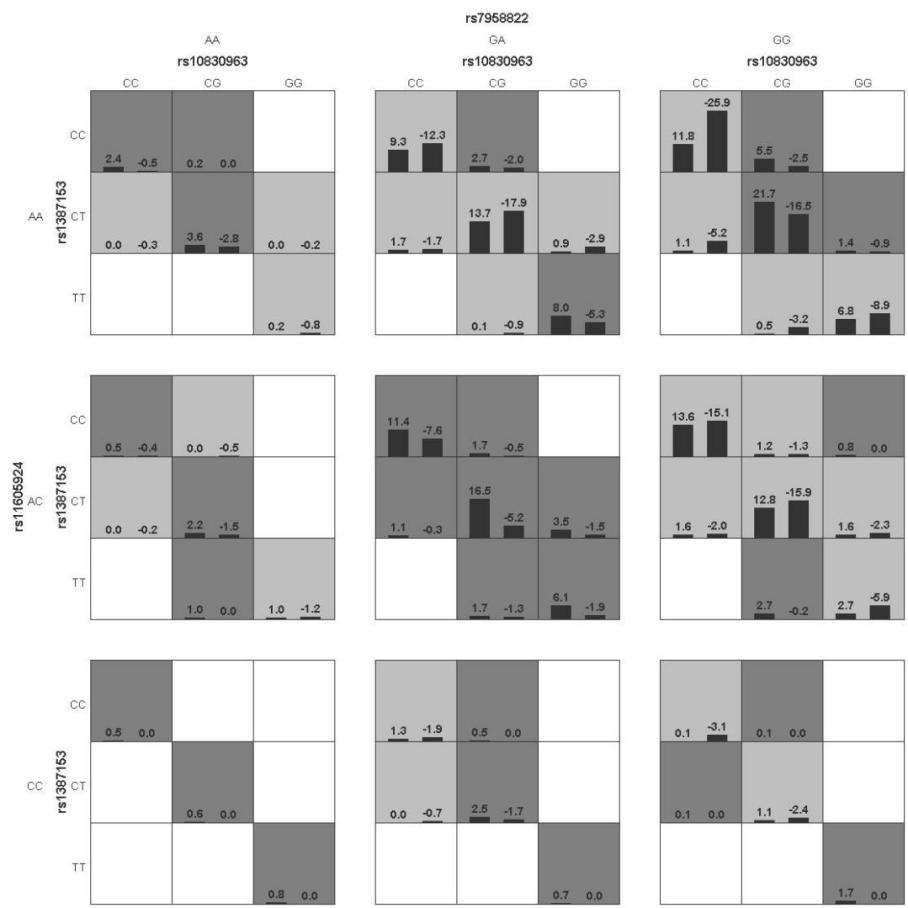


Note: Adjusted for age, marital status, per capita monthly household income, education level, smoking status, drinking status, salt taste preference, physical activity level, DASH score, liver function abnormalities, kidney function abnormalities, dyslipidemia, hypertension, family history of diabetes, CICRD, and occupational hazards (CO, noise, dust, and heat); Dark gray indicates the high-risk group, light gray indicates the low-risk group, and white indicates no samples.

Figure S3. Interaction model of four factors determined by GMDR


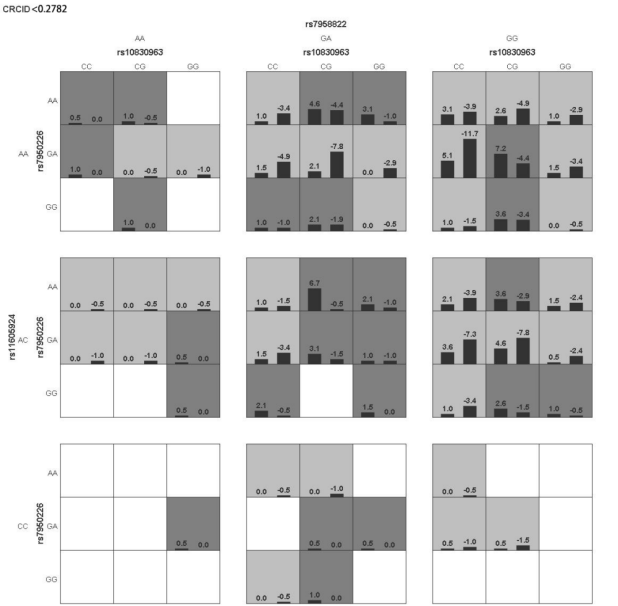

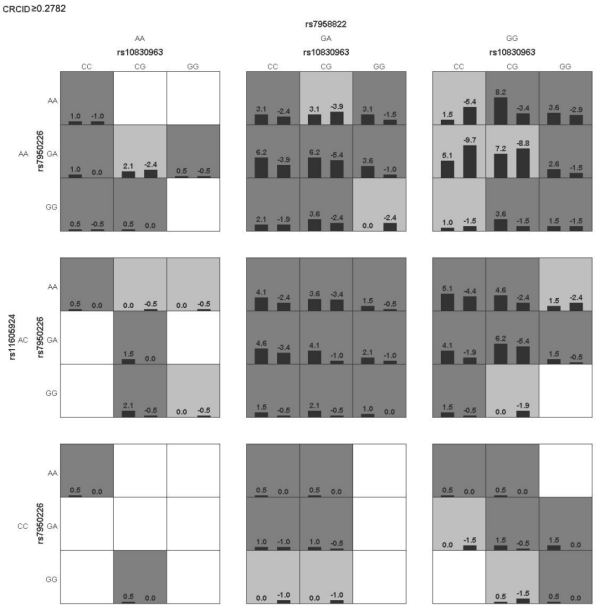


Note: Adjusted for age, marital status, per capita monthly household income, education level, smoking status, drinking status, salt taste preference, physical activity level, DASH score, liver function abnormalities, kidney function abnormalities, dyslipidemia, hypertension, family history of diabetes, CICRD, and occupational hazards (CO, noise, dust, and heat); Dark gray indicates the high-risk group, light gray indicates the low-risk group, and white indicates no samples.

Figure S4. Interaction model of five factors determined by GMDR
